# Role of PKC in the Regulation of the Human Kidney Chloride Channel ClC-Ka

**DOI:** 10.1038/s41598-020-67219-8

**Published:** 2020-06-24

**Authors:** Andrea Gerbino, Roberta De Zio, Daniela Russo, Luigi Milella, Serena Milano, Giuseppe Procino, Michael Pusch, Maria Svelto, Monica Carmosino

**Affiliations:** 1 Department of Sciences, University of Basilicata, Potenza, IT Italy; 2 National Research Council, Institute of Biomembrane and Bioenergetics, Bari, IT Italy; 3 National Research Council, Institute of Biophysics, Genova, IT Italy; 4 Department of Biosciences, Biotechnologies and Biopharamceutics, University of Bari, Bari, IT Italy

**Keywords:** Kidney, Protein transport, Ion channel signalling

## Abstract

The physiological role of the renal ClC-Ka/ClC-K1 channels is to confer a high Cl^-^ permeability to the thin Ascending Limb of Henle (tAL), which in turn is essential for establishing the high osmolarity of the renal medulla that drives water reabsorption from collecting ducts. Here, we investigated by whole-cell patch-clamp measurements on HEK293 cells co-expressing ClC-Ka (tagged with GFP) and the accessory subunit barttin (tagged with m-Cherry) the effect of a natural diuretic extract from roots of *Dandelion* (DRE), and other compounds activating PKC, such as ATP, on ClC-Ka activity and its membrane localization. Treatment with 400 µg/ml DRE significantly inhibited Cl^-^ currents time-dependently within several minutes. Of note, the same effect on Cl^-^ currents was obtained upon treatment with 100 µM ATP. Pretreatment of cells with either the intracellular Ca^2+^ chelator BAPTA-AM (30 μM) or the PKC inhibitor Calphostin C (100 nM) reduced the inhibitory effect of DRE. Conversely, 1 µM of phorbol meristate acetate (PMA), a specific PKC activator, mimicked the inhibitory effect of DRE on ClC-Ka. Finally, we found that pretreatment with 30 µM Heclin, an E3 ubiquitin ligase inhibitor, did not revert DRE-induced Cl^-^ current inhibition. In agreement with this, live-cell confocal analysis showed that DRE treatment did not induce ClC-Ka internalization. In conclusion, we demonstrate for the first time that the activity of ClC-Ka in renal cells could be significantly inhibited by the activation of PKC elicited by classical maneuvers, such as activation of purinergic receptors, or by exposure to herbal extracts that activates a PKC-dependent pathway. Overall, we provide both new information regarding the regulation of ClC-Ka and a proof-of-concept study for the use of DRE as new diuretic.

## Introduction

In the mammalian kidney, the loop of Henle plays a crucial role in the urine concentrating mechanism and consequently in blood pressure regulation. The descending part of the loop reabsorbs water while the ascending part, the thin and thick segments, mainly reabsorbs salt. The thin ascending limb (tAL) exhibits a large Cl^−^ permeability in both the apical as well as the basolateral membrane, which is mediated by the ClC-Ka channel in association with its accessory subunit barttin^1^. The resulting positive transepithelial voltage provides a driving force for paracellular Na^+^ reabsorption. In the thick ascending limb (TAL), the functional cooperation between the apical Na^+^/2Cl^−^/K^+^ cotransporter (NKCC2) and the inwardly rectifying potassium channel (ROMK1) with the basolateral complex of ClC-Kb/barttin results in a transcellular and electrogenic NaCl reabsorption. This salt movement toward the interstitium generates the cortico-medullary osmotic gradient needed for water reabsorption in the collecting ducts, where the vasopressin-dependent AQP2 translocation on the luminal membrane of collecting duct cells renders collecting ducts permeable to water.

This orchestrated plethora of membrane transporters regulating urine concentration and blood volume provides a number of present and possible future pharmacological targets for antihypertensive therapies. For instance, loop diuretics such as furosemide and bumetanide, inhibitors of the NKCC2 cotransporter, are among the most powerful antihypertensive drugs available to date. They act by increasing the volume of urine and the amount of excreted Na^+^ and are widely used for the clinical management of hypertension and edema^2^. However, considering growing concerns raised about adverse effects of classic diuretics such as thiazides and loop diuretics^3^, the availability of new synthetic, semi-synthetic or natural-derived (herbs and botanicals) diuretics have attracted considerable attention.

Among the renal transporters involved in the urine concentrating mechanisms described above, Cl^-^ channels ClC-Ka and ClC-Kb seem to be interesting targets for diuretic drugs (for review see^4^). In fact, two newly synthetized benzofuran derivatives, MT-189 and RT-93, with micromolar affinity for ClC-Ka/Kb (K_D_ < 10 μM), increased urine flow and significantly reduced blood pressure when orally fed to rats^5^. ClC-Ka and ClC-Kb are the human homologs of the isoforms in rodent, ClC-K1 and ClC-K2, respectively, with which they share the localization within renal tubules. ClC-Ka is expressed along both the apical and basolateral membranes in thin ascending limb (tAL) whereas ClC-Kb is expressed in the basolateral membrane of the TAL and cortical collecting ducts^6,7^. Both channels need barttin, a two-transmembrane protein, as an accessory subunit for proper function^1^. In addition, ClC-Ka, ClC-Kb and barttin are co-expressed in marginal cells in the inner ear^1^. Loss of function of ClC-Kb induces Bartter’s syndrome type III characterized by hypokalaemic alkalosis with salt wasting and low blood pressure^8^. ClC-K2 KO mice exhibit a phenotype that is similar to Bartter’s syndrome^9,10^ confirming that rodent ClC-K2 is the functional homolog of human ClC-Kb. In contrast, ClC-K1 KO mice exhibit a phenotype resembling Nephrogenic Diabetes Insipidus (NDI), characterized by dramatic polyuria associated with low urine osmolality^11,12^. Furthermore, ClC-K1 KO mice failed to concentrate urine after 24-h water deprivation or intraperitoneal injection of dDAVP^11^. These findings suggested that ClC-Ka and ClC-Kb have different physiological functions in the kidney, with ClC-Ka contributing mostly to the high osmolarity of the medulla and ClC-Kb retrieving most of the chloride that is left in the urine, making ClC-Ka, rather than ClC-Kb, a very interesting target for diuretics.

Very few studies have been conducted on the modulation of ClC-Ka activity. An important aspect in this regard is that ClC-Ka (as well as ClC-Kb), can only be functionally expressed together with the accessory subunit barttin which supports the exit of the channel from the endoplasmic reticulum, stimulates its insertion into the surface membrane and mediates its membrane removal through an ubiquitin-mediated mechanism^13,14^. Other regulatory mechanisms identified so far for both ClC-K channels are the inhibition at acidic extracellular pH and the activation at high extracellular Ca^2+^^13,15,16^, levels. Recently it has been shown that palmitoylation of barttin is necessary for activation of plasma membrane-inserted ClC-K/barttin channels^17^.

In a previous study, we found that non-toxic doses of a hydroethanolic Dandelion Root Extract (DRE) mobilize intracellular Ca^2+^ and activate PLC in HEK293 cells^18^. Interestingly, when extracts of dandelion were administered by gastric gavage to rats, a significant diuretic effect, paralleled by a weight loss, was obtained^19^. Moreover, different fractions of dandelion extracts slightly increased the final urine volume in mice^20^. Finally, in a pilot study on healthy people, daily administration of a dandelion extract increased significantly both the frequency of urination and the excretion ratio^21^. Motivated by this, we tested DRE on heterologously expressed ClC-Ka/barttin and found that the extract is able to significantly inhibit the activity of the ClC-Ka/barttin complex by a PKC mediated-mechanism. Furthermore, we found that DRE affected ClC-Kb activity in a similar manner but did affect neither NKCC2 nor AQP2 phosphorylation in our cell models, thus suggesting a specific effect of this diuretic herb *in vivo* on ClC-K activity. Neither barttin ubiquitination, nor channel internalization were involved in the inhibitory effect of DRE on ClC-Ka activity. Of note, the same inhibitory effect on ClC-Ka activity was elicited by purinergic receptors stimulation and by direct PKC activation. At the best of our knowledge here we reported the first evidence regarding the regulation of ClC-Ka by a PKC-associated signaling pathway with several physiological and translational implications.

## Results

### DRE time-dependently inhibits the Cl^−^ current in HEK293 cells co-expressing ClC-Ka and barttin

For all the functional experiments we used a dose of DRE (400 µg/mL) that we previously characterized and tested in terms of maximal tolerated concentration and ability to mobilize intracellular Ca^2+^ and activate phospholipase C (PLC) in HEK293 cells^18^.

**Figure 1:**
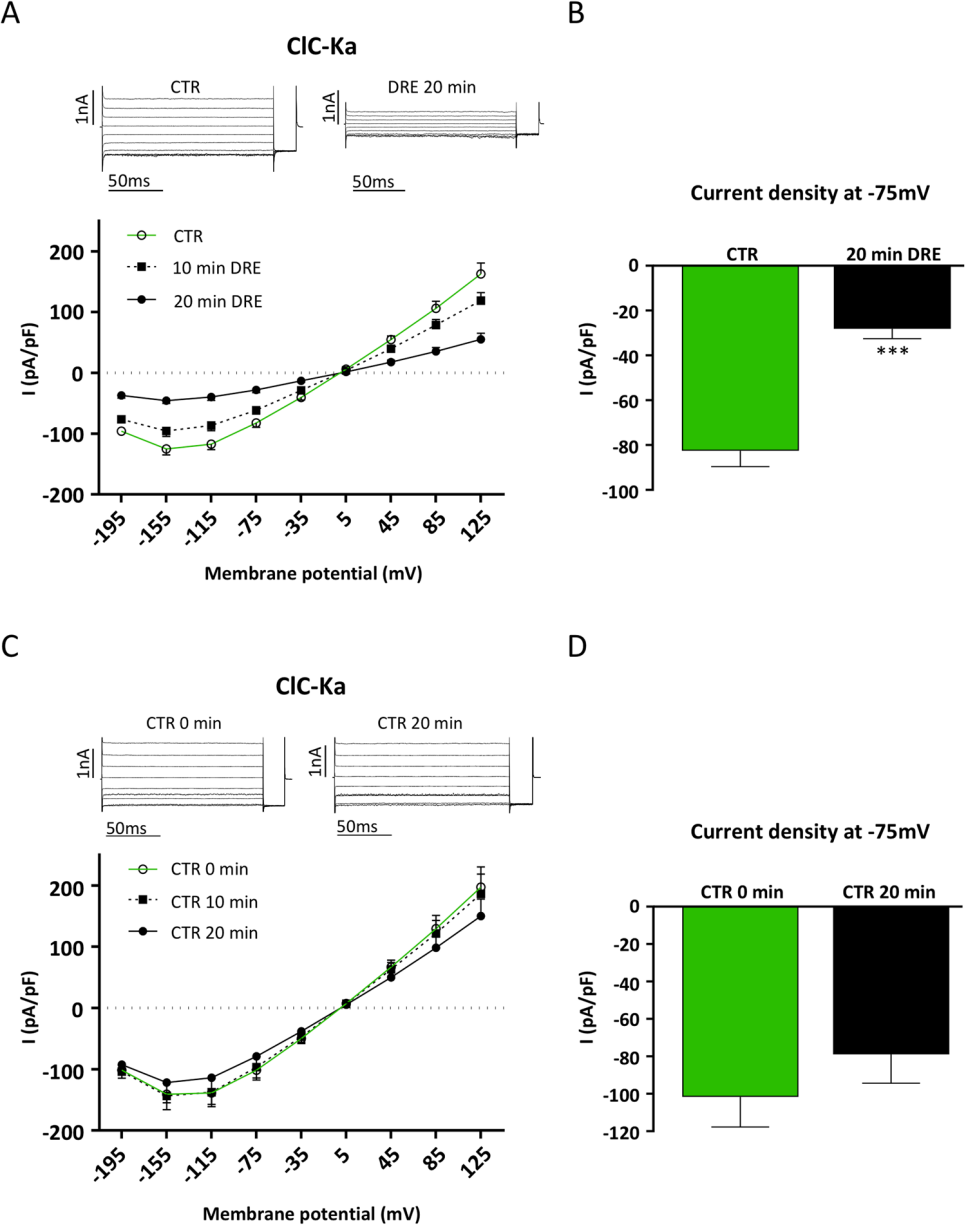
DRE inhibits Cl^−^ currents in ClC-Ka/barttin co-transfected HEK293 cells. (**A**) Upper panel. Representative whole-cell Cl^−^ currents recordings obtained in the same cell before (CTR) and after 20 min perfusion with DRE (DRE). Lower panel. Average current density/voltage relationship recorded in control condition (green line, open circles, CTR) and after perfusion for 10 (black dotted line, filled square, 10 min DRE) and 20 min (black line, filled circles, 20 min DRE) with 400 µg/mL DRE. Current data were normalized to the cell capacitances. Each point represents the average current for each voltage applied obtained from 6 cells. (**B**) Current density at −75 mV extracted from the *I/V* plot in control condition (CTR, green bar) or after 20 min perfusion with DRE (20 min DRE, black bar). (**C**) Upper panel. Representative whole-cell Cl^−^ currents recordings obtained in the same cell before (CTR) and after 20 min perfusion only with the Ringer’s solution (CTR 20 min). Lower panel. Average current density/voltage relationship recorded in Ringer’s solution at time 0 (green line, open circles, CTR 0 min) and after perfusion for 10 (black dotted line, filled square, CTR 10 min) and 20 min (black line, filled circles, CTR 20 min) with Ringer’s solution. (**D**) Current density at −75 mV extracted from the *I/V* plot in Ringer’s solution at time 0 (CTR 0 min, green bar) or after 20 min perfusion with Ringer’s solution (CTR 20 min, black bar). *** P < 0.001.

Figure 1A (Upper panel) reports representative Cl^−^ currents recorded from the same ClC-Ka/barttin expressing HEK293 cell, at the start of the whole-cell recording, i.e. shortly after break-in, (left, CTR) and after 20 min exposure to DRE (right, DRE), respectively. The lower panel shows average *I/V* plots illustrating the mostly ohmic voltage-dependence of ClC-Ka/barttin mediated currents with a slight decrease at most negative voltages, resulting in the well-known hook-shaped appearance of the *I-V* curve^22^. Importantly, 400 µg/mL DRE treatment time-dependently reduced Cl^−^ currents at both negative and positive potentials (10 min DRE, 20 min DRE). When we plotted values of Cl^−^ currents at −75 mV we found that 20 min DRE induced a strong and significant inhibition of the current intensity recorded in the same cell in control condition (from −82 ± 7.1 pA/pF to −28 ± 4.4 pA/pF, N = 6 cells) (Fig. 1B).

When we repeated the same experimental protocol for the evaluation of ClC-Ka activity in the absence of DRE addition (CTR 0 min), no significant changes in Cl^−^ currents at both negative and positive potentials were recorded during 20 min perfusion with Ringer’s solution (CTR 10 min and CTR 20 min) as shown by the representative ClC-Ka current traces and the average *I/V* plot (Fig. 1C). Plotting Cl^−^ currents at −75 mV (Fig. 1D) showed that 20 min perfusion with Ringer’s solution did not significantly change the current intensity recorded in the same cell in control condition (from −101.65 ± 16.1 pA/pF to −78,97 ± 15.42 pA/pF, N = 5 cells).

### DRE inhibits also the activity of ClC-Kb while other renal transporters involved in the urine concentrating mechanisms are unaffected

To verify whether the effect of 400 µg/mL DRE was specific for ClC-Ka, we investigated its effect on ClC-Kb activity when the channel was co-expressed with the accessory subunit barttin in HEK293.

**Figure 2:**
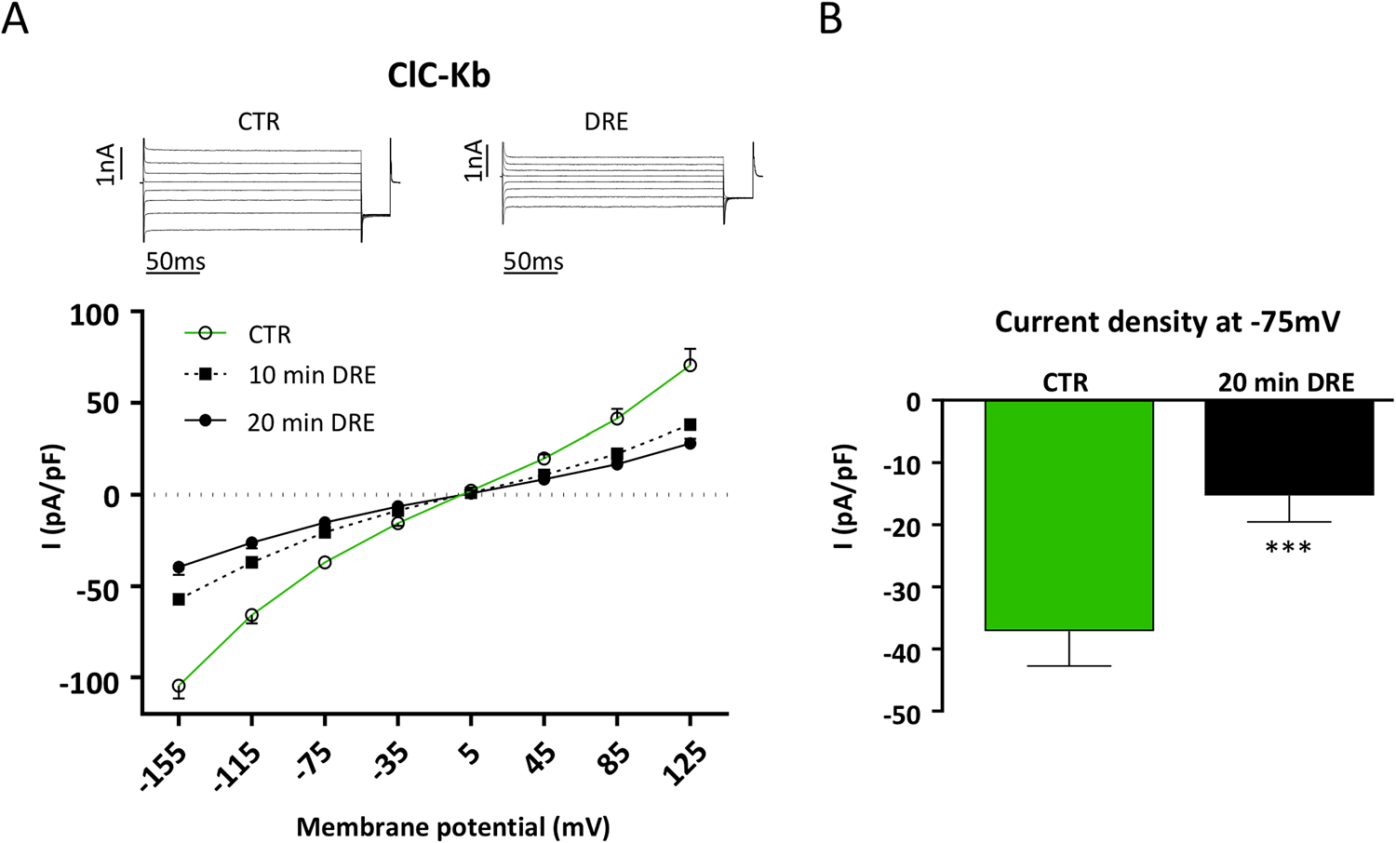
DRE effects on ClC-Kb activity in HEK293 cells. (**A**) Upper panel. Representative whole-cell ClC-Kb currents recordings obtained in the same cell before (CTR) and after 20 min perfusion with DRE (DRE). Lower panel. Average current density/voltage relationship recorded in control condition (green line, open circles, CTR) and after perfusion for 10 (black dotted line, filled square, 10 min DRE) and 20 min (black line, filled circles, 20 min DRE) with 400 µg/ml DRE. Current data were normalized to the cell capacitances. Each point represents the average current for each voltage applied obtained from 6 cells. (**B**) Current density at −75 mV extracted from the *I/V* plot in control conditions (CTR, green bar) or after 20 min perfusion with DRE (20 min DRE, black bar). ***P < 0.001.

As shown in Figure 2A, ClC-Kb currents exhibit a characteristic bi-directional rectification with whole-cell current amplitudes smaller than for ClC-Ka/barttin (Fig. 2A) as also reported by others^23^. The effect of DRE on ClC-Kb was similar to that observed for ClC-Ka; Cl^−^ currents were reduced time-dependently at both negative and positive potentials (10 min DRE, 20 min DRE). When we summarized values of ClC-Kb currents at −75 mV we found that 20 min DRE induced a strong and significant inhibition of the current intensity recorded in the same cell in control condition (−37.1 ± 2.8 pA/pF to −15.25 ± 2.1 pA/pF; N = 4 cells, Fig. 2B).

Moreover, we investigated the effect of DRE on the regulation of other transporters involved in the urine concentrating mechanisms such as NKCC2 and AQP2. The phosphorylation rate of NKCC2 (pNKCC2) in both basal and stimulated conditions, as index of its activation, was semi-quantified by western blotting in mouse kidney slices using an antibody that specifically recognizes the regulatory phospho-threonines 96 and 101^24^, required for NKCC2 activity^25,26^. Treatment with 400 µg/mL DRE did not affect the amount of pNKCC2 both under basal conditions and upon FK stimulation compared to slices not exposed to DRE (Supplementary Figure 1A, CTR, FK, +DRE). Similarly, we assessed the effect of DRE on AQP2 phosphorylation in mouse kidney slices, since AQP2 phosphorylation upon Vasopressin or FK stimulation is the key event required for AQP2 translocation from intracellular storage vesicles to the apical membrane which causes an increase in water permeability of renal principal cells^27,28^. We found a significant increase in the levels of phosphorylated AQP2 (pAQP2) independent of the pre-incubation with DRE (Supplementary Figure 1B, CTR, FK, +DRE). These data suggest that DRE neither inhibits NKCC2 activation in TALs nor AQP2 translocation in collecting ducts.

### The effect of DRE on ClC-Ka is mediated by the activation of PKC pathway

**Figure 3:**
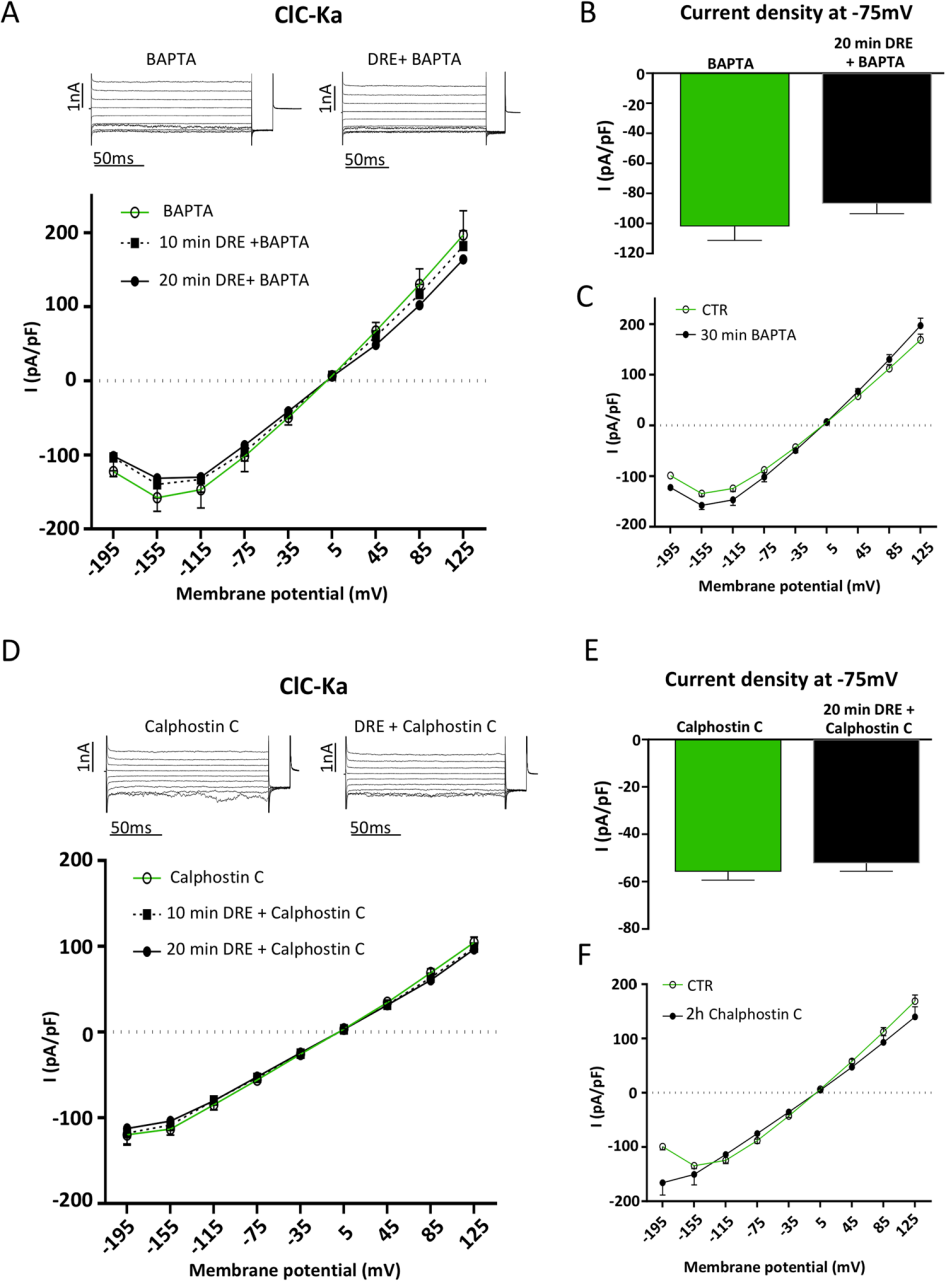
Effect of DRE on ClC-Ka activity is mediated by PKC. (**A**) Upper panel. Representative whole-cell Cl^-^ currents recordings obtained in the same BAPTA-pretreated cell before (BAPTA) and after 20 min perfusion with DRE (DRE + BAPTA). Lower panel. Average current density/voltage relationship recorded under BAPTA pretreatment before (green line, open circles, BAPTA) and after perfusion for 10 (black dotted line, filled square, 10 min DRE + BAPTA) and 20 min (black line, filled circles, 20 min DRE + BAPTA) with 400 µg/mL DRE. Current data were normalized to the cell capacitances. Each point represents the average current for each voltage applied obtained from 4 cells. (**B**) Current density at −75 mV extracted from the *I/V* plot under BAPTA pretreatment before (BAPTA, green bar) or after 20 min perfusion with DRE (20 min DRE + BAPTA, black bar). P = n.s. (**C**) Average current-density/voltage relationship recorded in control condition (green line, open circles, CTR) and after 30 min pretreatment with BAPTA (black line, filled circles, 30 min BAPTA). Current data were normalized to the cell capacitance. Each point represents the average current for each voltage applied obtained from either 20 (CTR) or 5 (30 min BAPTA) cells. (**D**) Upper panel. Representative whole-cell Cl^-^ currents recordings obtained in the same Calphostin C-pretreated cell before (Calphostin C) and after 20 min perfusion with DRE (DRE + Calphostin C). Lower panel. Average current density/voltage relationship recorded under Calphostin C pretreatment before (green line, open circles, Calphostin C) and after perfusion for 10 (black dotted line, filled square, 10 min DRE + Calphostin C) and 20 min (black line, filled circles, 20 min DRE + Calphostin C) with 400 µg/mL DRE. Each point represents the average current for each voltage applied obtained from 5 cells. (**E**) Current density at −75 mV extracted from the *I/V* plot under Calphostin C pretreatment before (Calphostin C, green bar) or after 20 min perfusion with DRE (20 min DRE + Calphostin C, black bar). (**F**) Average current-density/voltage relationship recorded in control condition (green line, open circles, CTR) and after 2 h pretreatment with Calphostin C (black line, filled circles, 2 h Calphostin C). Current data were normalized to the cell capacitance. Each point represents the average current for each voltage applied obtained from either 20 (CTR) or 8 (2 h Calphostin C) cells. P = n.s.

While dissecting the effect of DRE on Ca^2+^ signaling, we demonstrated that DRE-induced Ca^2+^ release from the ER is a consequence of the activation of the Gαq/PLC/IP3 pathway^18^ suggesting the activation of the conventional PKC, referred downstream in this signaling cascade as follow: PLC splits phosphatidylinositol into IP3 and diacylglycerol (DAG), the latter involved in the activation of the membrane-bound PKC together with intracellular Ca^2+ 29^. To evaluate whether the effect of DRE on ClC-Ka was dependent to the activation of conventional PKC pathway, we performed DRE experiments in the presence of either the Ca^2+^ chelator BAPTA or PKC inhibitor Calphostin C. As shown in Figure 3A, the inhibitory effect on ClC-Ka activity recorded after 20 min of exposure to 400 µg/mL DRE was abolished at each time point when the cells were treated with BAPTA. When we summarized values of ClC-Ka currents at −75 mV in the presence of BAPTA, we found that 20 min DRE did not induce any significant inhibition of the current intensity recorded in the same cell before the exposure to the herbal extract (−102 ± 9.2 pA/pF to −86.8 ± 6.6 pA/pF, N = 5 cells) (Fig. 3B). To evaluate the direct effect of BAPTA pre-treatment on ClC-Ka mediated currents we compared whole cell recordings in Ringer’s solution (CTR) with recordings obtained, in separate experiments, right after the end of BAPTA pre-treatment (30 min BAPTA). As shown in the *I/V* plots in Figure 3C, no differences were reported in ClC-Ka activity after the exposure to BAPTA at any voltages (CTR, 30 min BAPTA, N = 20 for CTR, N = 5 for 30 min BAPTA). Currents densities at −75 mV extracted from this analysis were reported in Supplementary Figure 2A.

Then, since PKC acts downstream the DRE-induced intracellular Ca^2+^ rise, we directly blocked PKC activity using Calphostin C at nanomolar concentration. This drug specifically inhibits PKC activity by competing at its binding site for DAG^30^. As shown in Figures 3D, 2h pretreatment with 100 nM Calphostin C reverted the effect induced by the exposure to 400 μg/mL DRE after 10 and 20 min, suggesting a direct involvement of PKC on ClC-Ka inhibition induced in these cells by DRE. When we summarized values of ClC-Ka currents at −75 mV in the presence of Calphostin C we found that 20 min DRE did not induce any significant inhibition of the current density recorded in the same cell before the exposure to the herbal extract (Fig. 3E, from −55.82 ± 3.55 pA/pF to −52.22 ± 3.43 pA/pF, N = 4 cells). To evaluate whether the prolonged (2 h) pre-treatment with Calphostin C could *per se* influence Cl^−^ currents, we directly compared ClC-Ka whole cell recordings in Ringer’s solution (CTR) with recordings obtained, in separate experiments, right after the end of Calphostin C pre-treatment (2 h Calphostin C). As shown in the *I/V* plot in Figure 3F, Calphostin C did not change the overall *I/V* plot when compared to control conditions (CTR, 2 h Calphostin C, N = 20 for CTR and N = 8 for 2 h Calphostin C) except for the lack of the hook-like appearance (between −195 mV and −155 mV) possibly related to a small off-target effect of the drug. Currents densities at −75 mV extracted from this analysis were reported in Supplementary Figure 2B.

### Stimulation of purinergic receptors with ATP inhibits ClC-Ka activity

**Figure 4:**
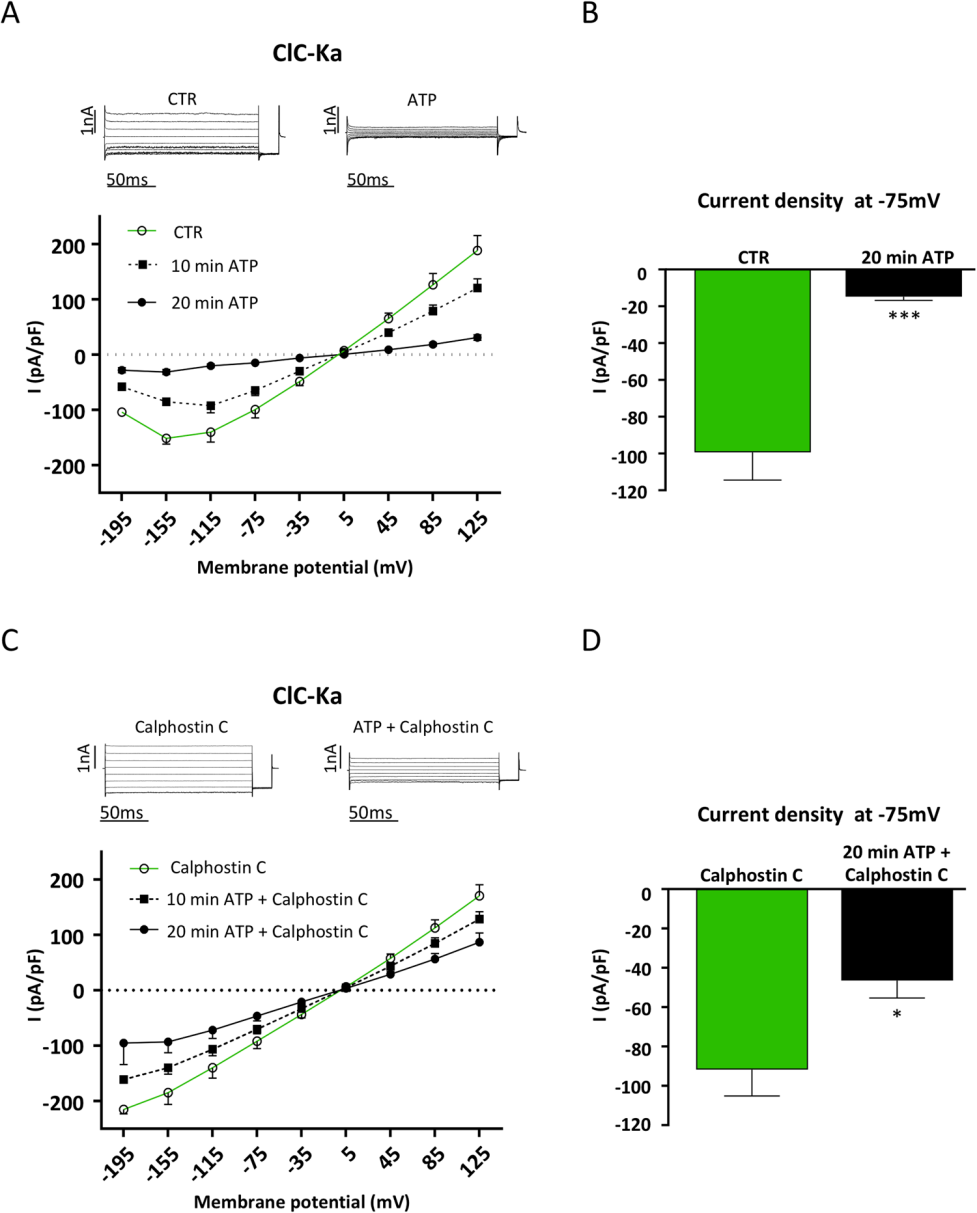
Stimulation of purinergic receptors with ATP inhibits ClC-Ka. (**A**) Upper panel. Representative whole-cell Cl^-^ currents recordings obtained in the same cell before (CTR) and after 20 min perfusion with ATP (ATP). Lower panel. Average current density/voltage relationship recorded in control condition (green line, open circles, CTR) and after perfusion for 10 (black dotted line, filled square, 10 min ATP) and 20 min (black line, filled circles, 20 min ATP) with 200 µM ATP. Current data were normalized to the cell capacitances. Each point represents the average current for each voltage applied obtained from 4 cells. (**B**) Current density at −75 mV extracted from the *I/V* plot in control conditions (CTR, green bar) or after 20 min perfusion with ATP (20 min ATP, black bar). ***P < 0.001. (**C**) Upper panel. Representative whole-cell Cl^-^ currents recordings obtained in the same Calphostin C-pretreated cell before (Calphostin C) and after 20 min perfusion with ATP (ATP + Calphostin C). Lower panel. Average current density/voltage relationship recorded under Calphostin C pretreatment before (green line, open circles, Calphostin C) and after perfusion for 10 (black dotted line, filled square, 10 min ATP + Calphostin C) and 20 min (black line, filled circles, 20 min ATP + Calphostin C) with 200 µM ATP. Current data were normalized to the cell capacitances. Each point represents the average current for each voltage applied obtained from 5 cells. B) Current density at −75 mV extracted from the *I/V* plot under Calphostin C pretreatment before (Calphostin C, green bar) or after 20 min perfusion with ATP (20 min ATP + Calphostin C, black bar). *P < 0.05.

Since HEK293 cells endogenously express purinergic receptors associated to the activation of Ca^2+^/PKC pathway^31,32^, we tested whether ATP was also able to inhibit the activity of ClC-Ka in HEK293 cells. Interestingly, as shown in Figure 4A, when HEK293 cells transfected with ClC-Ka and barttin were stimulated with 200 µM ATP, Cl^−^ currents were significantly reduced. Quantification of Cl^−^ currents at −75 mV shows that 20 min ATP induced a strong and significant inhibition of the current density recorded in the same cell in the absence of the agonist (from −99.35 ± 15.13 pA/pF to −14.75 ± 1.99 pA/pF; N = 4 cells) (Fig. 4B).

To verify whether the effect of purinergic receptors activation on ClC-Ka currents was also mediated by the activity of PKC, we stimulated cells with ATP after 100 nM Calphostin pretreatment. As shown in Figure 4C, Calphostin C pretreatment partially reverted the inhibitory effect induced by ATP on ClC-Ka activity after 10 and 20 min. When we plotted Cl^−^ currents at −75 mV we found that in Calphostin C pretreated cells, ATP still induced a residual inhibition of the current density recorded in the same cell before the addition of the agonist (from −91.7 ± 13.4 pA/pF to −46.43 ± 8.8 pA/pF; N = 4 cells) (Fig. 4D).

### ClC-Ka activity is inhibited by the PKC activator PMA

**Figure 5:**
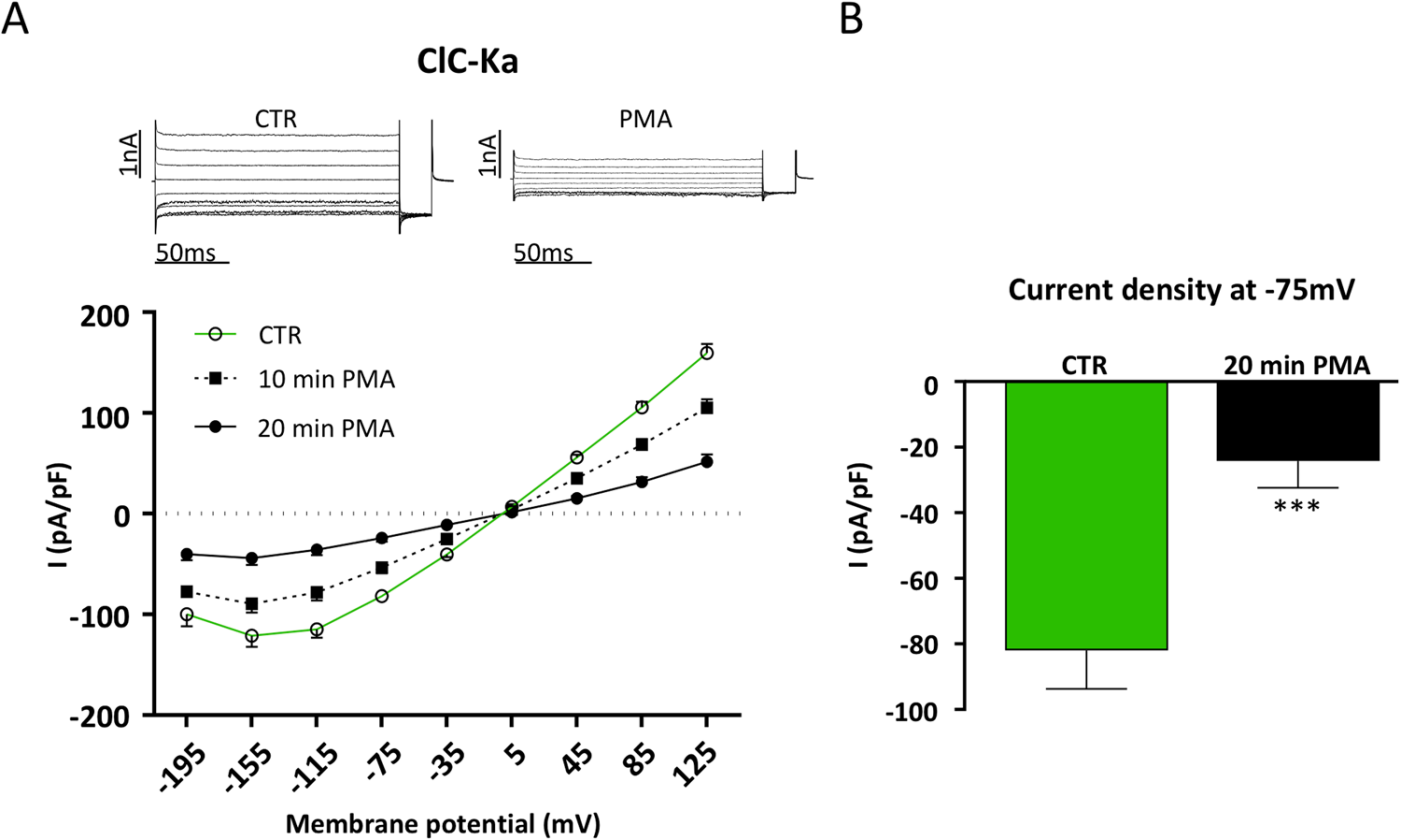
ClC-Ka is inhibited by PMA treatment. (**A**) Upper Panel. Representative whole-cell Cl^−^ currents recordings obtained in the same cell before (CTR) and after 20 min perfusion with PMA (PMA). Lower panel. Average current density/voltage relationship recorded in control condition (green line, open circles, CTR) and after perfusion for 10 (black dotted line, filled square, 10 min PMA) and 20 min (black line, filled circles, 20 min PMA) with 1 µM PMA. Current data were normalized to the cell capacitances. Each point represents the average current for each voltage applied obtained from 5 cells. (**B**) Current density at −75 mV extracted from the *I/V* plot in control conditions (CTR, green bar) or after 20 min perfusion with PMA (20 min PMA, black bar). ***P < 0.001.

Since purinergic receptors can be coupled to multiple pathways in renal cells (for review see^33^) we checked whether the direct activation of PKC may be able, by itself, to recapitulate the effect of ATP and DRE. To this end we used Phorbol 12-myristate 13-acetate (PMA), which is a specific and widely used PKC activator. As shown in the *I/V* plot of Figure 5A, acute exposure to 1 µM PMA significantly and time dependently reduced the Cl^−^ currents recorded in the same ClC-Ka expressing HEK293 cells in control condition. As shown in Figure 5B the Cl^−^ current density recorded at −75 mV dropped from −81 ± 5.2 pA/pF (CTR) to −24 ± 3.6 pA/pF after 20 min of exposure to PMA (N = 5 cells).

### DRE-mediated ClC-Ka inhibition does not involve channel endocytosis or channel ubiquitination

**Figure 6:**
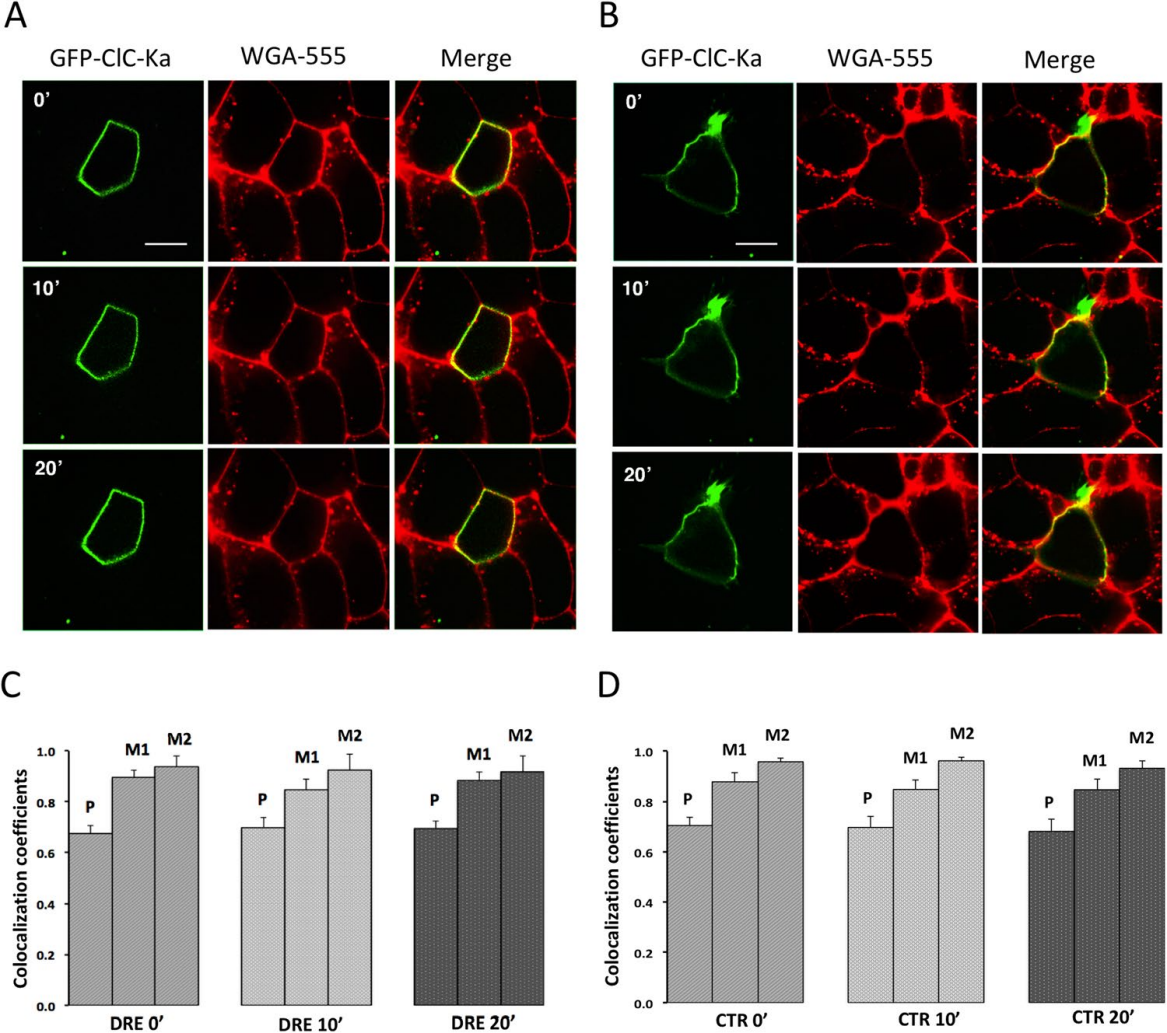
DRE-mediated ClC-Ka inhibition does not involve endocytosis. (**A**) Live spinning-disk confocal microscopy evaluation of ClC-Ka membrane localization in HEK293 cells co-transfected with GFP-ClC-Ka/untagged barttin and labeled with the plasma membrane marker WGA-555. ClC-Ka and WGA images were collected on the same focal plane before (0′) and after 10 min (10′) and 20 min (20′) perfusion with DRE. (**B**) Live spinning-disk confocal microscopy evaluation of ClC-Ka membrane localization in HEK293 cells co-transfected with GFP-tagged ClC-Ka/untagged barttin and labeled with the plasma membrane marker WGA-555. ClC-Ka and WGA images were collected on the same focal plane during perfusion with Ringer’s solution at time 0 (0′) and after 10 min (10′) and 20 min (20′). (**C**) Histograms showing the Pearson’s correlation coefficient (P) and the Mander’s overlap coefficients (M1 and M2) of the two channels (WGA-555 and GFP-ClC-Ka) calculated before (DRE 0′) and after 10 min (DRE 10′) and 20 min (DRE 20′) perfusion with DRE. M1 indicates WGA overlapping with ClC-Ka while M2 indicates ClC-Ka overlapping with WGA. The analysis was performed on 15 cells. P = n.s. (**D**) Histograms showing the Pearson’s correlation coefficient (P) and the Mander’s overlap coefficients (M1 and M2) of the two channels (WGA-555 and GFP-ClC-Ka) calculated during perfusion with Ringer’s solution at time 0 (CTR 0′) and after 10 min (CTR 10′) and 20 min (CTR 20′). M1 indicates WGA overlapping with ClC-Ka while M2 indicates ClC-Ka overlapping with WGA. The analysis was performed on 14 cells. P = n.s.

It has been reported that the ubiquitin ligase Nedd4–2, a member of the HECT domain-containing E3 ligases, mediates ClC-Ka/barttin internalization, acting through the ubiquitination of the PY motif in the barttin subunit^34^. It is also known that PKC-mediated Nedd4–2 activation may mediate the ubiquitination and internalization of several membrane transporters^35–37^. To investigate whether the activation of the PKC pathway triggered by DRE induces ClC-Ka/barttin internalization, we performed live spinning-disk confocal microscopy experiments using a wheat germ agglutinin (WGA) tagged with Alexa Fluor 555 (AF555) as marker of the plasma membrane. In these experiments HEK293 cells where simultaneously transfected with two plasmids encoding ClC-Ka-GFP and untagged barttin. Images of cells with a predominant plasma membrane localization of ClC-Ka were acquired at different time points, upon perfusion with DRE (Fig. 6A, DRE, 0′, 10′, 20′) or with the Ringer’s solution alone (Fig. 6B, CTR 0′, 10′, 20′). ClC-Ka-GFP localization at the plasma membrane is visualized as its co-localization with WGA-555 (yellow signal, Merge). The Pearson's correlation coefficient (P) and the Mander’s overlap coefficients (M1 and M2) were calculated in individual cells for GFP-ClC-Ka and WGA-555 at the same time-point described above (see methods for details). Both coefficients range between −1 (perfect negative correlation) to +1 (perfect positive correlation) with 0 meaning no correlation. As showed in the histogram in Figure 6C,D, both coefficients are informative that a significant colocalization between GFP-ClC-Ka and WGA-555 occurred at each time point and that it did not significantly change neither during exposure to DRE nor after exposure to Ringer solution. ClC-Ka-GFP signal at the plasma membrane was also quantified at each time points using a region of interest (ROI) where ClC-Ka colocalized with WGA-555. No significant changes in GFP-ClC-Ka fluorescence intensity at the plasma membrane were found at any time points (Supplemental Fig. 3).

**Figure 7:**
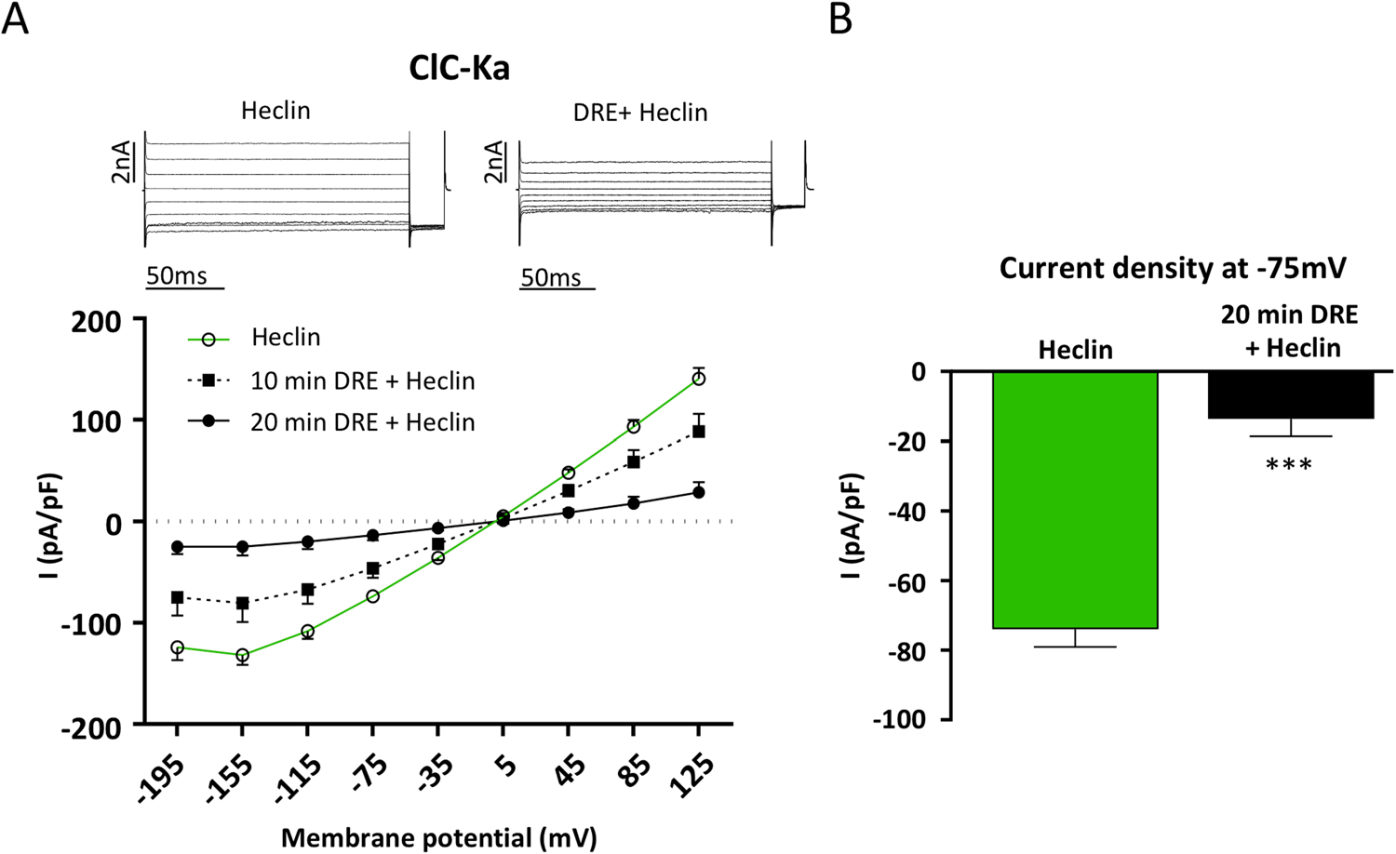
DRE-mediated ClC-Ka inhibition does not involve ubiquitination. (**A**) Upper panel. Representative whole-cell Cl^-^ currents recordings obtained in the same Heclin-pretreated cell before (Heclin) and after 20 min perfusion with DRE (DRE + Heclin). Lower panel. Average current density/voltage relationship recorded under Heclin pretreatment before (green line, open circles, Heclin) and after perfusion for 10 (black dotted line, filled square, 10 min DRE + Heclin) and 20 min (black line, filled circles, 20 min DRE + Heclin) with 400 µg/ml DRE. Current data were normalized to the cell capacitances. Each point represents the average current for each voltage applied obtained from 6 cells. (**B**) Current density at −75 mV extracted from the *I/V* plot under Heclin pretreatment before (Heclin, green bar) or after 20 min perfusion with DRE (20 min DRE + Heclin, black bar). ***P < 0.001.

It has been observed that, at least in oocytes, barttin ubiquitination by Nedd4–2 significantly inhibits Cl^−^ current through ClC-Ka while slightly reducing ClC-Ka membrane localization only at very high concentrations^34^. We thus investigated whether barttin ubiquitination might be involved in the inhibition of ClC-Ka activity independently of ClC-Ka/Barttin endocytosis. To this end we tested the effect of Heclin, a HECT ligase inhibitor, on Cl^−^ currents in ClC-Ka/barttin expressing HEK293 cells preincubated for 2 h with 30 µM of Heclin and then treated with 400 µg/mL DRE in the continuous presence of Heclin. It has been already demonstrated that in these experimental conditions Heclin is able to abolish overall protein ubiquitination in HEK293 cells without compromising cell viability^38^. As shown in Figure 7, Heclin did not revert DRE-induced Cl^−^ currents inhibition in ClC-Ka/Barttin-expressing HEK293 cells. Treatment with 400 µg/mL of DRE still induced a significant reduction in Cl^−^ channel activity after 10 and 20 min of DRE exposure regardless of Heclin treatment. As shown in Figure 7B, in the presence of Heclin, the Cl^−^ current density recorded at −75 mV (time 0, Heclin) was significantly reduced after 20 min exposure to DRE (from −73 ± 5.1 pA/pF to −13 ± 5 pA/pF; N = 6 cells), thus suggesting that the ClC-Ka inhibition is independent from channel ubiquitination.

## Discussion

The electrophysiological and functional properties of ClC-K channels remain imperfectly known. The only regulatory properties identified so far for this class of Cl^−^ channels are the inhibition at acid and at very basic extracellular pH, the activation at high extracellular Ca^2+^^13,15,16,39^ and a possible regulation by palmitoylation of barttin^17^. The physiological impact of the regulation by extracellular Ca^2+^ remains however uncertain since both ClC-Ka and ClC-Kb are relatively insensitive to extracellular Ca^2+^ variation in the physiological range^40,41^. The dependence on extracellular pH on the other hand may have functional implication during alkalosis and acidosis.

In contrast, information about the regulation of ClC-K channels by intracellular messengers is incomplete and debated. It has been reported that the activity of ClC-K1 in the mouse cortical TAL is stimulated by intracellular cAMP^42^. However, this regulation was not confirmed for the human ortholog ClC-Ka when expressed in HEK293 cells^16^ (our data, not shown). Lourdel *et al*. demonstrated that Cl^−^ transport across the basolateral membrane of mouse DCT, presumably through ClC-K2 channels, was not modified when tubules were pre-incubated with FK or when catalytically active PKA subunit was applied to the intracellular side of excised patches^43^. In contrast, in the DCT the channel was significantly inhibited by PMA^43^. The modulation by PKC activation was confirmed for the human ortholog ClC-Kb expressed in HEK293 cells^16^. Indeed, the scenario described so far, suggests that native mouse ClC-K1 (but not ClC-K2) is stimulated by cAMP/PKA pathways. Evidence regarding down-regulation by PKC-associated pathways is available only for ClC-K2/ ClC-Kb.

In this work we showed for the first time how the activity of PKC, modulated by bioactive compounds (DRE), a physiological agonist (ATP) or a synthetic drug (PMA), can be responsible of ClC-Ka inhibition in HEK293 cells. We found that an herbal extract, that we previously demonstrated to be coupled to a Ca^2+^/PLC pathway^18^, inhibits ClC-Ka mediated Cl^−^ currents in HEK293 cells. Interestingly, DRE-induced inhibition on ClC-Ka activity was completely abolished in the presence of the PKC inhibitor Calphostin C, which at nanomolar concentrations specifically inhibit PKC but not other kinases^29,30^. Although we noticed that 2 h pretreatment with Calphostin C changes *per se* the ClC-Ka open probability at hyperpolarized potentials (as shown by the absence of the typical hook-shaped *I/V* plot in Fig. 3F), we also found that 2 h Calphostin C treatment did not affect the overall Cl^−^ current amplitudes when compared to untreated cells (Fig. 3F) corroborating our conclusions on the experiments reported in Figure 3D.

Moreover, the effect of the extract was also abolished in the presence of the Ca^2+^ chelator BAPTA. Although we cannot exclude a direct effect of intracellular Ca^2+^ on ClC-Ka upon DRE treatment, our experimental evidence depicts a scenario in which intracellular Ca^2+^ rather triggers a downstream pathway coupled with the activation of Ca^2+^-sensitive PKC isozymes.

Furthermore, the activation of purinergic receptors by ATP led to a similar inhibition on ClC-Ka when compared with the effect induced by the herbal extract. However, blockage of PKC by Calphostin C significantly reduced, but not completely reverted, the effect induced by ATP on ClC-Ka activity. The partial inhibitory effect of Calphostin C upon ATP treatment can be explained by two different hypotheses. One possibility is that ATP could inhibit ClC-Ka acting also through a different pathway. HEK293 cells endogenously express at least three different P2Y receptors (P2Y1, P2Y2 and P2Y11)^31,32^ and one of them, P2Y11, could be coupled with other intracellular players, thus we cannot exclude that PKC activation is somehow more consistent than after exposure to DRE. Accordingly, Calphostin C could not be potent enough to completely revert ATP-induced inhibition of ClC-Ka. A second possibility is that Calphostin C-insensitive PKC isozymes are involved in the ATP action in HEK293 cells. It is known that HEK293 cells express a plethora of PKC isozymes^44,45^ including atypical DAG-insensitive PKC isozymes^46^, which are known to be less sensitive to Calphostin C^47^.

In agreement with the absence of DRE (or ATP)-induced ClC-Ka inhibition when PKC in blocked by Calphostin C, direct activation of PKC by PMA significantly inhibited ClC-Ka activity to the same extent as for DRE.

Of note, in our hands, exposure to DRE also inhibits ClC-Kb activity. This evidence is in agreement with the reported down-regulation of ClC-Kb in response to PMA-induced PKC activation in HEK293 cells^16^. Interestingly, Lourdel and collaborators found that ClC-K2 (the rodent homolog of ClC-Kb) was only inhibited by PMA and not by the increase in intracellular Ca^2+^ obtained by ionomycin in perfused mouse kidney tubules^43^, supporting the hypothesis that ClC-K channels are inhibited by PKC but not by the increase in intracellular Ca^2+^ *per se*. Another member of the large family of ClC channels, ClC-1, is selectively expressed in skeletal muscle and downregulated upon PKC activation^48^. Interestingly, Pedersen *et al*. revealed that the PKC involved in the inhibition of ClC-1 activity was activated by the Ca^2+^ released from the sarcoplasmic reticulum, clearly indicating the involvement of conventional Ca^2+^-sensistive PKC isoforms^49^. It has been postulated that PKC acts presumably by phosphorylating one or more residues in the intracellular C-terminal region of the ClC-1 channel^50^.

On the other hand, how PKC modulates ClC-Ka (and ClC-Kb) is still an open question. Here we demonstrated that neither ubiquitination nor endocytic internalization of the ClC-Ka/barttin complex is involved in the Cl^−^ current inhibition induced by DRE. We hypothesize that PKC could modulate the activity of the channel phosphorylating either ClC-Ka or Barttin subunit since both proteins contain consensus sites for PKC.

Very recently, the zinc finger DHHC-type containing 7 (DHHC7) palmitoyl-acyltransferase has been identified to induce barttin palmitoylation and ClC-K channel activation^51^. Thus, PKC may modulate the activity of DHHC7 on putative phosphorylation sites. Indeed, we cannot exclude that PKC is only a piece of a more complex puzzle in the mechanisms involved in ClC-Ka inhibition. Addressing these open and fascinating questions on the PKC-dependent modulation of ClC-Ka will deepen their understanding at a molecular level in the near future.

From a physiological point of view, the regulation of ClC-Ka by PKC-associated pathways may have several implications. Interestingly, Bailey *et al*. demonstrated that basolateral administration of ATP on isolated rat tAL induced a robust intracellular Ca^2+^ increase also in the absence of extracellular Ca^2+^^52^. Moreover, the authors demonstrated that purinergic receptors of the P2Y2 subtype, known to be coupled to PKC activation^53^, might mediate this effect. Although the functional role of the ATP/P2Y axis in tAL has not been studied, the inhibitory role of this axis in renal salt reabsorption in TAL and water reabsorption in CD has been largely demonstrated^54^. In this context, the inhibitory effect of ATP through PKC activation on ClC-Ka activity in tAL would be in line with the demonstrated role of the purinergic system in the mammalian nephron where it antagonizes urine concentrating mechanisms^54^.

Interestingly, vasopressin V1a receptors have been identified in tAL of the mammalian nephron^55^. Although there is still no evidence regarding their functional role in this nephron segment, V1a receptors have been associated to both activation of PLC/PKC^56^ and reduction in the antidiuretic effects of vasopressin in rat collecting ducts^57^. These pieces of evidence could suggest that the inhibition of ClC-Ka through V1a-dependent activation of PKC might be the mechanism underlying reduction of water reabsorption in tAL.

From a pharmacological point of view the results shown here suggest that the diuretic effect of DRE *in vivo*^19,21^ may be mediated at least partially by its action on ClC-Ka and ClC-Kb (or ClC-K1 and ClC-K2 in rodents). More *in vivo* studies are definitively needed for better understanding how extracts from dandelion may affect the urinary excretion of several electrolytes. We cannot exclude, in fact, that the inhibition of ClC-Kb activity mediated by DRE may cause a type III Bartter like syndrome if DRE was administered *in vivo*. However, some findings reported that DRE and the well-characterized ClC-K inhibitors preferentially act on ClC-K1 rather then ClC-K2 when administered *in vivo*. Accordingly, one on of the few studies reporting the diuretic effect of dandelion extract in rats reported also a blunted natriuretic effects of the extract^20^. In agreement with this latter finding, benzofuran derivatives such as MT-189 and RT-93, blocking both ClC-K1 and ClC-K2 channels, induced significant diuretic effects and lowered the systemic blood pressure without affecting electrolyte balance when administered *in vivo* in rats, suggesting that *in vivo* they were acting as inhibitors of ClC-K1 rather ClC-K2 channels^5^.

In conclusion, here we showed for the first time that the activation of PKC can modulate, in intact cells, the activity of the renal human ClC-Ka channel, adding another piece of knowledge in the complex scenario of signal pathways involved in regulation of renal transporters.

Moreover, although further studies are needed to identify the chemical identity and the pharmacology of the molecule/s in DRE responsible for the effect of the extract on ClC-Ka and ClC-Kb activities, we provided a ‘proof-of-concept study’ on the development of dandelion extracts as new natural diuretics acting on ClC-K channels.

## Materials and Methods

### Cells and constructs

HEK293 cells were transiently transfected with the pcDNA3.1 plasmid encoding for ClC-Ka tagged with GFP at the N-terminal tail and with the pcDNA3.1 plasmid encoding for barttin with m-Cherry tagged at the C-terminal tail. Starting from an untagged construct of ClC-Ka in pcDNA3.1^16^, EGFP was fused N-terminally with PCR and overlapping primers, inserted using an upstream pcDNA3.1 NotI site and an internal BglII site in the ClC-Ka sequence, resulting in the following sequence at the junction between EGFP and ClC-Ka, with EGFP sequence underlined:

… aag ggt tct gga atg gag gag ttg gtg …

… Lys Gly Ser Gly Met Glu Glu Leu Val …

M-cherry-tagged barttin construct was kindly donated by Dr. Alessandra Picollo, National Research Council, Institute of Biophysics, Genova, IT.

GFP- tagged ClC-Kb and untagged barttin were kindly provided by Prof. Antonella Liantonio, Department of Pharmacy-Drug Sciences, University of Bari, IT.

Transfection was performed using Lipofectamin 2000 reagent (Thermo Fisher Scientific, Waltham, MA, USA), according to the manufacturer’s protocol.

### Kidney slices: preparation and treatment

Kidney slices were prepared and treated as previously described with some modification regarding the treatment^47^. Briefly, adult C57BL/6 J male mice were anesthetized with an intraperitoneal injection of tri- bromoethanol (250 mg/kg) and euthanized by cervical dislocation. Kidneys were quickly removed, and sections of approximately 250 μm were cut using a McILWAIN Tissue Chopper (Ted Pella, Inc., Redding, Ca, USA). Kidney slices were incubated at 37 °C for 15 min in Dulbecco’s modified Eagle’s medium-GlutaMAX (Thermo Fisher Scientific, Waltham, MA, USA) containing 20 mM HEPES sodium salt. Medium was previously pre-equilibrated in a 5% CO_2_ incubator. After equilibration, slices were stimulated for 30 min at 37 °C either with DRE (400 μg/ml) or with the vehicle alone (DMSO), in the same medium. Another set of slices was stimulated for 30 min with DRE (400 μg/ml) either alone or followed by 40 min incubation with Forskolin (FK, 100 µM). Control slices were either stimulated with Forskolin (FK, 100 µM) or with the vehicle alone. Treated slices were then subjected to western blotting analysis.

### Tissue fractionation and immunoblotting

Samples from tissues were processed as follows: cells or slices were lysed in ice-cold antiphosphatase buffer^58^ and sonicated for 15 sec. Slices were lysed for 60 additional min on ice. Unsolubilized material was pelleted by centrifugation at 13,000 g for 30 min at 4 °C. Supernatants were separated by standard SDS-PAGE and analyzed by western blotting. Samples were resolved on 7.5% or 12% Mini-PROTEAN TGX Stain-Free Precast Gels (Bio-Rad Laboratories, Hercules, CA, U.S.A) according to protein size. After blocking with 3% bovine serum albumin in TRIS buffer saline-tween 20 (TBS-T), blots were incubated overnight at 4 °C with the following antibodies in blocking buffer: antibody anti-p-NKCC2 (kindly provided by Prof. Biff Forbush from the Department of Cellular and Molecular Physiology, Yale University, New Haven, CT, USA, dil. 1:500) and anti-p-AQP2 (Rabbit affinity-purified polyclonal antibody against rat phosphorylated AQP2 at serine 256 were produced as previously described^59^, anti-NKCC2 (dil.1:500, cat. #3562 P, Millipore, Billerica, MA, USA). Membranes were washed and incubated with horseradish peroxidase–conjugated secondary antibody. Negative controls with secondary antibodies alone were performed (not shown). Target proteins were revealed with an enhanced chemiluminescent detection system superSignal West Pico Chemiluminescent Substrate (Thermo Fisher Scientific, Waltham, MA, USA). Chemiluminescence was detected with a Chemidoc XRS detection system. Acquisition and analysis of blot images were performed with Image Lab Software 6.01 (https://www.bio-rad.com/en-us/product/image-lab-software?ID = KRE6P5E8Z). Bands densitometry was carried out after normalization for the total protein loading (Stain-Free technology, Bio-Rad Laboratories, Hercules, CA, U.S.A.).

### Imaging by confocal microscopy

HEK293 cells transiently transfected with ClC-Ka-GFP and barttin-mCherry constructs were fixed with 4% paraformaldehyde in PBS for 20 min, washed in PBS and mounted for confocal imaging. Confocal images were obtained with a confocal laser-scanning fluorescence microscope Leica TSC-SP2 (Leica Microsystem, Wetzlar, Germany).

For monitoring endocytosis, HEK293 cells seeded on glass coverslip (15 mm ø) and expressing ClC-Ka-GFP and untagged barttin were incubated for 10 min with 0.5 µg/ml Wheat Germ Agglutinin, Alexa Fluo 555 Conjugate (W32464, Thermo Fisher Scientific, Waltham, MA, USA) in PBS at room temperature. Cells were mounted on an open-top perfusion chamber and constantly perfused either with the Ringer alone or with DRE 400 µg/ml for 20 min The set-up is composed of a Nikon Eclipse TE 2000-U fluorescent microscope equipped with a 40×/1.30 N.A. fluor objective (Nikon) and a spinning-disk confocal setup (CrEST CARV II, assembled by CRISEL Instruments, https://www.crisel-instruments.it). GFP-ClC-Ka fluorescence was excited with a green laser (SVL-473–0200 at 473 nm of excitation wavelength) and recorded at 520 nm emission wavelength. WGA-555 was excited at 555 nm using a mercury lamp light and the emission collected at 585 nm. Excitation light was projected through 1000 pinholes (Ø 70 µm) using a CREST CARVII spinning disk (http://www.bioimagingsolutions.com/Crest/CARVII/CrestCARVII_Home.html). Both fluorophores emission wavelengths were collected by a Photometrics Cool Snap HQ camera (1392 × Z1040 imaging pixels) and digitalized with the MetaMorph 7.8.6.0 (ID 6828, https://www.moleculardevices.com/products/cellular-imaging-systems/acquisition-and-analysis-software/metamorph-microscopy#gref). Five confocal images (16 bit) where collected for each run (with one or two transfected cells) starting from time 0 (control condition in Ringer’s solution) and every 5 min for 20 min, either with or without DRE addition. Images were then background subtracted and used for the quantitative evaluation of the ClC-Ka fluorescence intensity at the plasma membrane selecting region of interest (ROI) where the channel colocalized with WGA using Fiji (https://imagej.net/Fiji). Figures only shows the analysis performed at the same time points used to evaluate ClC-Ka activity in the electrophysiological experiments. Cells in which the exogenous expression of the channel was predominantly located within the cytosol where excluded from the colocalization analysis (WGA-ClC-Ka). The same images were used to evaluate the Pearson correlation coefficient (P) and the Mander’s overlap coefficients (M1 and M2) for GFP-ClC-Ka and WGA-555 using the Fiji plugin JACoP (Just Another Colocalisation Plugin) according developer’s instructions^60^. The analysis was performed using ROI selecting around individual whole cells. Cells in close contact where analyzed within the same image. The appropriate threshold was automatically calculated by the plugin for the two channels (red and green) of every image.

### Electrophysiological recordings

Whole-cell recordings were performed at room temperature on HEK293 transiently expressing either GFP-ClC-Ka or GFP-ClC-Kb chloride channel and the accessory subunit barttin-mCherry. Twenty-four hours after transfection, cells were detached and re-seeded at lower concentration on 35 mm plate pre-coated with Poly-L-Lys hydrobromide (P2636; Sigma, St. Louis, Missouri, U.S.A.) and used for recordings once adherent. During the experiments the cells were perfused (at 2 ml/minute) with a bath solution containing (in mM): 140 NaCl, 5 KCl, 1 MgCl_2_, 10 HEPES, 1 CaCl_2_, 5 Glucose; pH 7.4. Bath Ringer’s solution was modified whenever needed adding different compounds depending on the experimental approach. The effect of continuous exposure to 400 µg/ml DRE on ClC-Ka/ClC-Kb activity was tested in the same HEK293 cell first recording Cl^−^ currents before DRE addition in the Ringer’s solution (CTR) and secondly after 10 and 20 min after DRE addition. The same experimental approach was used when we tested the effects of either 200 µM ATP (Sigma, St. Louis, Missouri, U.S.A.) or 1 µM PMA (Sigma, St. Louis, Missouri, U.S.A.) on ClC-Ka activity. Furthermore, control experiments in Ringer’s solution were performed using the same experimental design except for drugs addition, thus whole-cells recordings were performed starting at time 0 after 10 and 20 min of perfusion in Ringer’s solution.

When we assessed the effects of 30 µM BAPTA-AM (B6769, Thermofisher Scientific, Waltham, MA, U.S.A.), 100 nM Calphostin C (Sigma, St. Louis, Missouri, U.S.A.) and 30 µM Heclin (Sigma, SML1396, St. Louis, Missouri, U.S.A.) on DRE-induced ClC-Ka inhibition, transfected HEK293 cells were pretreated at 37 °C for 30 min, 2 h and 2 h, respectively. In addition, drugs were continuously perfused throughout the experiments with a (PPS2) MCS Peristaltic Pump Perfusion System (Warner Instruments, Harvard Bioscience, Holliston, MA, USA).

35 mm bottom dish with ClC-Ka/barttin (or ClC-Kb/barttin) transfected HEK293 cells were then mounted in a Quick Exchange Platform (QE-1, Warner Instruments, Harvard Bioscience, Holliston, MA, USA) and housed on the microscope stage of an upright Olympus BX51WI (Olympus, Tokyo, Japan). The sample was illuminated through a 40× water immersion objective (NA = 0.80) by monochromatic lights (at 488 and 585 nm). The emitted fluorescence was passed through correspondent dichroic mirrors and filtered at 510 nm or 610 (both Omega Optical, Brattleboro, VT), respectively. HEK293 cells expressing both ClC-Ka-GFP (or ClC-Kb-GFP) and barttin-mCherry were selected according green/red fluorescence at the plasma membrane.

Borosilicate patch pipettes were pulled to obtain a tip resistance of 3–5 MΩ with a P-1000 Pipette puller (Sutter Instrument, Novato, CA, USA) and filled with a pipette solution containing (in mM): 120 NaCl, 2 MgCl_2_, 5 EGTA, 10 HEPES; pH 7.4.

Patch pipettes were connected to a Multiclamp 700B amplifier (Axon CNS-Molecular Devices, Sunnyvale, CA, USA) interfaced with the Axon Digidata 1500 (Axon Instrument-Molecular Devices, Sunnyvale, CA, USA). Currents were sampled at 10 kHz and low-pass filtered at 5 kHz. AxoScope 10.4 (Molecular Devices, Sunnyvale, CA, USA) and pClamp 10.4 (Molecular Devices, Sunnyvale, CA, USA) were used to acquire and analyze the data. Current recordings were normalized on cell capacitance and expressed as current density (pA/pF).

The voltage dependence of chloride current amplitude was determined using the following voltage clamp protocol for HEK293 cells expressing ClC-Ka /barttin complex: voltage steps (40 mV steps for 150 ms) between −195 mV and +125 mV starting from the holding potential of 0 mV; pulses ended with a tail pulse to −120 mV for 20 ms^61^. The voltage clamp protocol applied for HEK293 cells expressing ClC-Kb /barttin complex was: voltage steps (40 mV steps for 150 ms) between −155 mV and +125 mV starting from the holding potential of 0 mV; pulses ended with a tail pulse to −120 mV for 20 ms^61^.

In both cases the evoked currents were measured at each step in pA and plotted against the voltage obtaining the IV plots of the currents. Data in the IV plots are presented as mean ± SEM.

### Statistical analysis

All statistical analyses were performed with GraphPad Prism 7.0 (https://www.graphpad.com/). Two-way ANOVA test was used to evaluate the effect of treatment and exposure time on ClC-Ka current as expressed in the *I/V* plots. Unless otherwise stated, histograms of current density at −75 mV in control condition and after 20 min of treatment are extracted from the *I/V* plots such as the statistical analysis. Statistical analysis of western blot data was performed using a one-way ANOVA test (with Dunnett’s multicomparisons post-hoc analysis). Statistical analysis of live imaging confocal data was calculated by one-way ANOVA (Tukey’s multiple comparison test). A value of P < 0.05 was considered statistically significant.

## Supplementary information

Supplementary Information.

## References

[1] Jentsch TJ, Pusch M (2018). CLC Chloride Channels and Transporters: Structure, Function, Physiology, and Disease. Physiol. Rev..

[2] Ellison DH (2019). Clinical Pharmacology in Diuretic Use. Clin. J. Am. Soc. Nephrol..

[3] Testani JM, Chen J, McCauley BD, Kimmel SE, Shannon RP (2010). Potential effects of aggressive decongestion during the treatment of decompensated heart failure on renal function and survival. Circulation.

[4] Fong P (2004). CLC-K channels: if the drug fits, use it. EMBO Rep..

[5] Liantonio A (2012). *In-vivo* administration of CLC-K kidney chloride channels inhibitors increases water diuresis in rats: a new drug target for hypertension?. J. Hypertens..

[6] Jentsch TJ, Stein V, Weinreich F, Zdebik AA (2002). Molecular structure and physiological function of chloride channels. Physiol. Rev..

[7] Uchida S, Sasaki S (2005). Function of chloride channels in the kidney. Annu. Rev. Physio.l.

[8] Simon DB (1997). Mutations in the chloride channel gene, CLCNKB, cause Bartter’s syndrome type III. Nat. Genet..

[9] Grill A (2016). Salt-losing nephropathy in mice with a null mutation of the Clcnk2 gene. Acta Physiol. (Oxf).

[10] Hennings JC (2017). The ClC-K2 Chloride Channel Is Critical for Salt Handling in the Distal Nephron. J. Am. Soc. Nephrol..

[11] Matsumura Y (1999). Overt nephrogenic diabetes insipidus in mice lacking the CLC-K1 chloride channel. Nat. Genet..

[12] Liu W (2002). Analysis of NaCl transport in thin ascending limb of Henle’s loop in CLC-K1 null mice. Am. J. Physiol. Renal Physio.l.

[13] Estevez R (2001). Barttin is a Cl- channel beta-subunit crucial for renal Cl- reabsorption and inner ear K+ secretion. Nature.

[14] Scholl U (2006). Barttin modulates trafficking and function of ClC-K channels. Proc. Natl. Acad. Sci. USA.

[15] Gradogna A, Babini E, Picollo A, Pusch M (2010). A regulatory calcium-binding site at the subunit interface of CLC-K kidney chloride channels. J. Gen. Physiol..

[16] Imbrici P, Liantonio A, Gradogna A, Pusch M, Camerino DC (2014). Targeting kidney CLC-K channels: Pharmacological profile in a human cell line versus Xenopus oocytes. Bba-Biomembranes.

[17] Steinke KV (2015). Human CLC-K Channels Require Palmitoylation of Their Accessory Subunit Barttin to Be Functional. J. Biol. Chem..

[18] Gerbino, A. *et al*. Dandelion Root Extract Induces Intracellular Ca(2+) Increases in HEK293 Cells. *Int*. *J. Mol. Sci*. **19**, 10.3390/ijms19041112 (2018). 10.3390/ijms19041112 PMC5979456 29642457

[19] Racz-Kotilla E, Racz G, Solomon A (1974). The action of Taraxacum officinale extracts on the body weight and diuresis of laboratory animals. Planta Med..

[20] Hook I, McGee A, Henman M (1993). Evaluation of Dandelion for Diuretic Activity and Variation in Potassium Content. International Journal of Pharmacognosy.

[21] Clare BA, Conroy RS, Spelman K (2009). The diuretic effect in human subjects of an extract of Taraxacum officinale folium over a single day. J. Altern. Complement. Med..

[22] Fischer M, Janssen AG, Fahlke C (2010). Barttin activates ClC-K channel function by modulating gating. J. Am. Soc. Nephrol..

[23] Tan H, Bungert-Plumke S, Fahlke C, Stolting G (2017). Reduced Membrane Insertion of CLC-K by V33L Barttin Results in Loss of Hearing, but Leaves Kidney Function Intact. Front. Physiol..

[24] Flemmer AW, Gimenez I, Dowd BF, Darman RB, Forbush B (2002). Activation of the Na-K-Cl cotransporter NKCC1 detected with a phospho-specific antibody. J. Biol. Chem..

[25] Gimenez I, Forbush B (2005). Regulatory phosphorylation sites in the NH2 terminus of the renal Na-K-Cl cotransporter (NKCC2). *Am*. J. Physiol. Renal Physiol..

[26] Carmosino M (2011). NKCC2 is activated in Milan hypertensive rats contributing to the maintenance of salt-sensitive hypertension. Pflugers Arch..

[27] Fushimi K, Sasaki S, Marumo F (1997). Phosphorylation of serine 256 is required for cAMP-dependent regulatory exocytosis of the aquaporin-2 water channel. J. Biol. Chem..

[28] Procino G (2003). Ser-256 phosphorylation dynamics of Aquaporin 2 during maturation from the ER to the vesicular compartment in renal cells. FASEB J..

[29] Lipp, P. & Reither, G. Protein kinase C: the “masters” of calcium and lipid. *Cold Spring Harb. Perspect. Biol*. **3**, 10.1101/cshperspect.a004556 (2011). 10.1101/cshperspect.a004556 PMC3119906 21628429

[30] Kobayashi E, Nakano H, Morimoto M, Tamaoki T, Calphostin C (1989). UCN-1028C), a novel microbial compound, is a highly potent and specific inhibitor of protein kinase C. Biochem. Biophys. Res. Commun..

[31] Moore DJ (2001). Expression pattern of human P2Y receptor subtypes: a quantitative reverse transcription-polymerase chain reaction study. Biochim. Biophys. Acta.

[32] Tran E, Sun H, Fang Y (2012). Dynamic mass redistribution assays decode surface influence on signaling of endogenous purinergic P2Y receptors. Assay Drug Dev. Technol..

[33] Burnstock G, Evans LC, Bailey MA (2014). Purinergic signalling in the kidney in health and disease. Purinergic Signal.

[34] Embark HM (2004). Regulation of CLC-Ka/barttin by the ubiquitin ligase Nedd4-2 and the serum- and glucocorticoid-dependent kinases. Kidney Int..

[35] Vina-Vilaseca A, Bender-Sigel J, Sorkina T, Closs EI, Sorkin A (2011). Protein kinase C-dependent ubiquitination and clathrin-mediated endocytosis of the cationic amino acid transporter CAT-1. J. Biol. Chem..

[36] Vina-Vilaseca A, Sorkin A (2010). Lysine 63-linked polyubiquitination of the dopamine transporter requires WW3 and WW4 domains of Nedd4-2 and UBE2D ubiquitin-conjugating enzymes. J. Biol. Chem..

[37] Garcia-Tardon N (2012). Protein kinase C (PKC)-promoted endocytosis of glutamate transporter GLT-1 requires ubiquitin ligase Nedd4-2-dependent ubiquitination but not phosphorylation. J. Biol. Chem..

[38] Mund T, Lewis MJ, Maslen S, Pelham HR (2014). Peptide and small molecule inhibitors of HECT-type ubiquitin ligases. Proc. Natl. Acad. Sci. USA.

[39] Gradogna A, Pusch M (2013). Alkaline pH Block of CLC-K Kidney Chloride Channels Mediated by a Pore Lysine Residue. Biophys. J..

[40] Andrini O (2014). CLCNKB mutations causing mild Bartter syndrome profoundly alter the pH and Ca2+ dependence of ClC-Kb channels. Pflug. Arch. Eur. J. Phy..

[41] Gradogna, A. & Pusch, M. Molecular pharmacology of kidney and inner ear CLC-K chloride channels. Front. Pharmacol. **1**, UNSP 130 10.3389/fphar.2010.00130 (2010). 10.3389/fphar.2010.00130 PMC3153005 21833170

[42] Paulais M, Teulon J (1990). Camp-Activated Chloride Channel in the Basolateral Membrane of the Thick Ascending Limb of the Mouse Kidney. J. Membrane Biol..

[43] Lourdel S, Paulais M, Marvao P, Nissant A, Teulon J (2003). A chloride channel at the basolateral membrane of the distal-convoluted tubule: a candidate ClC-K channel. J. Gen. Physiol..

[44] Jackson A, Sedaghat K, Minerds K, James C, Tiberi M (2005). Opposing effects of phorbol-12-myristate-13-acetate, an activator of protein kinase C, on the signaling of structurally related human dopamine D1 and D5 receptors. J. Neurochem..

[45] Liu M (2017). Co-ordinated activation of classical and novel PKC isoforms is required for PMA-induced mTORC1 activation. PLoS One.

[46] Kajimoto, T. *et al*. Activation of atypical protein kinase C by sphingosine 1-phosphate revealed by an aPKC-specific activity reporter. *Sci. Signal* **12**, 10.1126/scisignal.aat6662 (2019). 10.1126/scisignal.aat6662 PMC6657501 30600259

[47] Mizuno K (1995). UCN-01, an anti-tumor drug, is a selective inhibitor of the conventional PKC subfamily. FEBS Lett..

[48] Chen MF, Jockusch H (1999). Role of phosphorylation and physiological state in the regulation of the muscular chloride channel ClC-1: a voltage-clamp study on isolated M. interosseus fibers. Biochem. Biophys. Res. Commun..

[49] Pedersen TH, Macdonald WA, de Paoli FV, Gurung IS, Nielsen OB (2009). Comparison of regulated passive membrane conductance in action potential-firing fast- and slow-twitch muscle. J. Gen. Physiol..

[50] Hsiao KM, Huang RY, Tang PH, Lin MJ (2010). Functional study of CLC-1 mutants expressed in Xenopus oocytes reveals that a C-terminal region Thr891-Ser892-Thr893 is responsible for the effects of protein kinase C activator. Cell Physiol. Biochem..

[51] Gorinski, N. *et al*. DHHC7-mediated palmitoylation of the accessory protein barttin critically regulates the functions of ClC-K chloride channels. *J. Biol. Chem*., 10.1074/jbc.RA119.011049 (2020). 10.1074/jbc.RA119.011049 PMC7196637 32184353

[52] Bailey MA (2000). Axial distribution and characterization of basolateral P2Y receptors along the rat renal tubule. Kidney Int..

[53] Erb L, Liao ZJ, Seye CI, Weisman GA (2006). P2 receptors: intracellular signaling. Pflug. Arch. Eur. J. Phy..

[54] Vallon V, Stockand J, Rieg T (2012). P2Y receptors and kidney function. Wiley Interdiscip. Rev. Membr. Transp. Signal.

[55] Imbert-Teboul M, Champigneulle A (1995). [Functional expression of vasopressin receptors V1a and V2 along the mammalian nephron]. C. R. Seances Soc. Biol. Fil..

[56] Wasilewski MA, Myers VD, Recchia FA, Feldman AM, Tilley DG (2016). Arginine vasopressin receptor signaling and functional outcomes in heart failure. Cell Signal.

[57] Ikeda M, Yoshitomi K, Imai M, Kurokawa K (1994). Cell Ca2+ response to luminal vasopressin in cortical collecting tubule principal cells. Kidney Int..

[58] Gerbino A (2016). Spilanthol from Acmella Oleracea Lowers the Intracellular Levels of cAMP Impairing NKCC2 Phosphorylation and Water Channel AQP2 Membrane Expression in Mouse Kidney. PLoS One.

[59] Christensen BM, Zelenina M, Aperia A, Nielsen S (2000). Localization and regulation of PKA-phosphorylated AQP2 in response to V(2)-receptor agonist/antagonist treatment. *Am*. J. Physiol. Renal Physiol..

[60] Bolte S, Cordelieres FP (2006). A guided tour into subcellular colocalization analysis in light microscopy. J. Microsc..

[61] Riazuddin S (2009). Molecular basis of DFNB73: mutations of BSND can cause nonsyndromic deafness or Bartter syndrome. *Am*. J. Hum. Genet..

