# Publisher Correction: Role of PKC in the Regulation of the Human Kidney Chloride Channel ClC-Ka

**DOI:** 10.1038/s41598-020-74040-w

**Published:** 2020-09-30

**Authors:** Andrea Gerbino, Roberta De Zio, Daniela Russo, Luigi Milella, Serena Milano, Giuseppe Procino, Michael Pusch, Maria Svelto, Monica Carmosino

**Affiliations:** 1 Department of Sciences, University of Basilicata, Potenza, IT Italy; 2 Institute of Biomembrane and Bioenergetics, National Research Council, Bari, IT Italy; 3 Institute of Biophysics, National Research Council, Genova, IT Italy; 4 Department of Biosciences, Biotechnologies and Biopharamceutics, University of Bari, Bari, IT Italy

Correction to: *Scientific Reports* 10.1038/s41598-020-67219-8, published online 24 June 2020

The Funding section in this Article was omitted. The Funding section should read:

“MC was supported by a grant from the Italian Research Ministry (n. ARS01_01081). MP was supported by a grant from the Fondazione AIRC per la Ricerca sul Cancro (grant # IG 21558) and by a grant from the Italian Research Ministry (PRIN 20174TB8KW).”

