# Supplementary material for: Role of PKC in the Regulation of the Human Kidney Chloride Channel ClC-Ka
Source: Sci Rep. 2020 Jun 24;10:10268. doi: 10.1038/s41598-020-67219-8 (PMC7314819; doi:10.1038/s41598-020-67219-8)
Supplement: Supplementary file 1 — Supplementary Information. [file 41598_2020_67219_MOESM1_ESM.pdf]

SUPPLEMENTARY INFORMATION

Role of PKC in the Regulation of the Human Kidney Chloride Channel ClC-Ka

Andrea Gerbino

Roberta De Zio

Daniela Russo

Luigi Milella

Serena Milano

Giuseppe Procino

Michael Pusch

Maria Svelto

Monica Carmosino

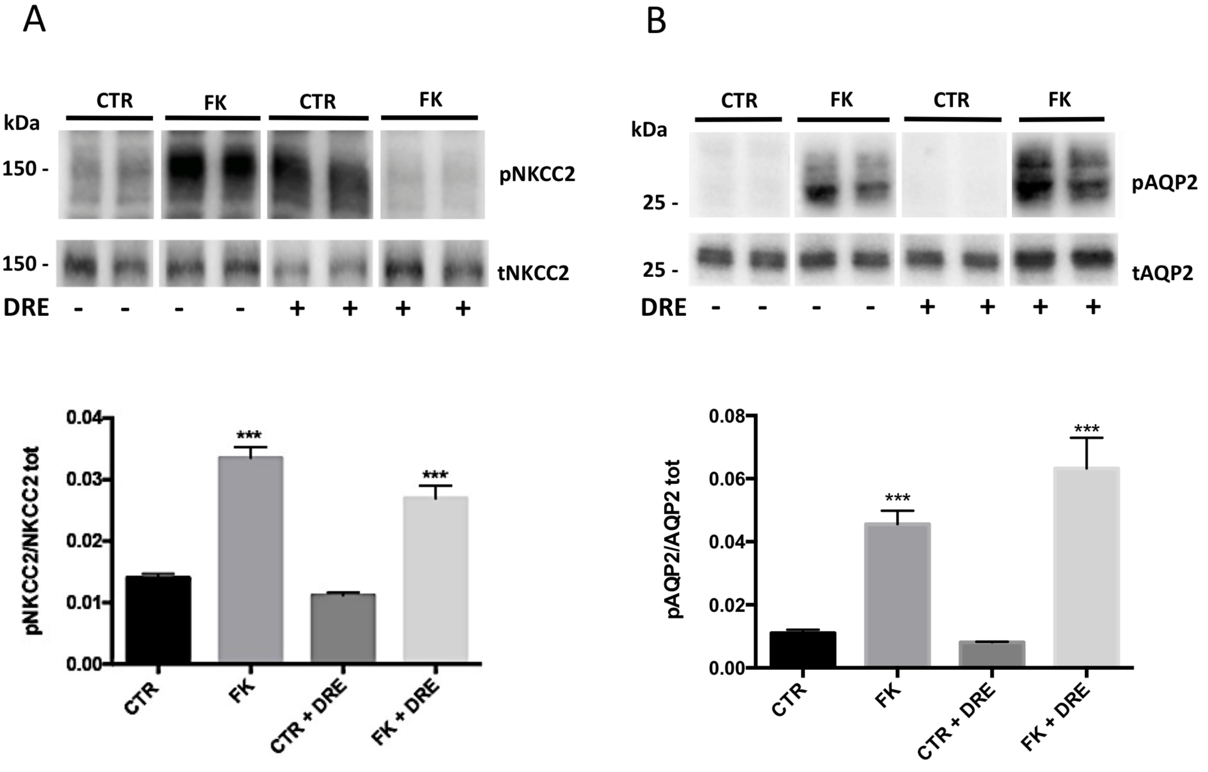

Supplementary Figure 1: A) Upper panel. Representative Western blot for the analysis of the expression of both phospho-NKCC2 (pNKCC2) and total NKCC2 (tNKCC2) in lysates from mouse kidney slices in control conditions (CTR) or stimulated with Forskolin (FK) in the presence of DRE where indicated (+,- DRE). Two bands of interest for each treatment (CTR and FK ±DRE) cropped from different parts of the same blot are shown separated by white spaces. Original blots are shown in Supplementary Figure 4. Lower panel. Densitometric analysis for the semiquantization of pNKCC2 normalized *vs* tNKCC2 in three independent experiments. Significance was calculated by one-way Anova test, ***P<0.001. B) Upper panel. Representative Western blot for the analysis of the expression of both phospho-AQP2 (pAQP2) and total AQP2 (tAQP2) in lysates from mouse kidney slices in control conditions (CTR) or stimulated with Forskolin (FK) in the presence of DRE where indicated (+,- DRE). Two bands of interest for each treatment (CTR and FK ±DRE) cropped from different parts of the same blot are shown separated by white spaces. Original blots are shown in Supplementary Figure 5. Lower panel. Densitometric analysis for the semiquantization of pAQP2 normalized *vs* tAQP2 in three independent experiments. Significance was calculated by one-way Anova test. ***P<0.001

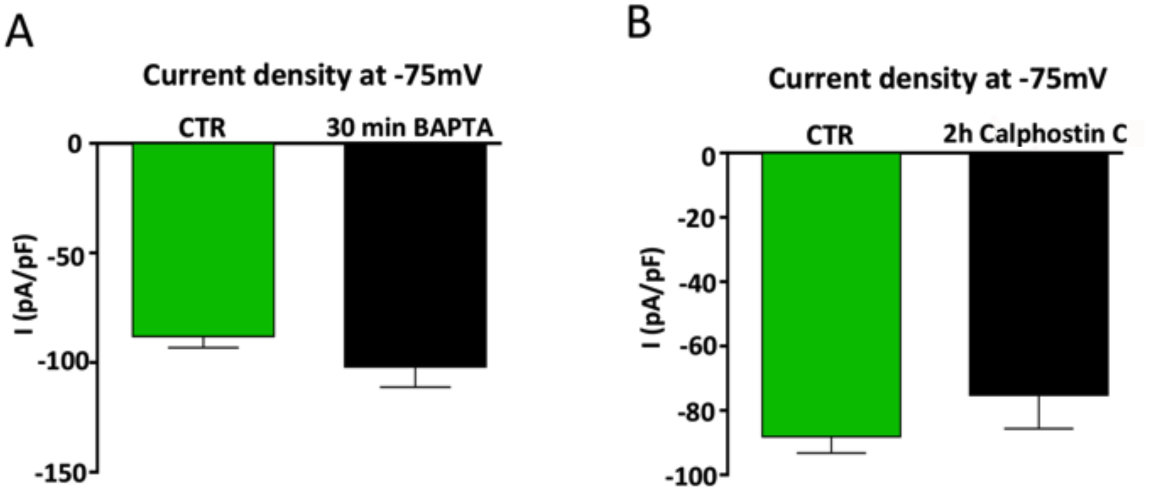

Supplementary Figure 2. Neither Bapta- nor Calphostin C pretreatment affect ClC-Ka current density at -75 mV. A) Current-density at -75 mV extracted from the *I/V* plot in control conditions (CTR, green bar, -88 ± 5.12 pA/pF) or after 30 min pretreatment with BAPTA (30 min BAPTA, black bar, -102 ± 9.23 pA/pF). These data sets were statistically analysed with a Student's T-Test for unpaired data. P=n.s. B) Current-density at -75 mV extracted from the *I/V* plot in control conditions (CTR, green bar, -88 ± 5.12 pA/pF) or after 2 h pretreatment with Calphostin C (2h Calphostin C, black bar, -75,16 ± 10,48 pA/pF). These data sets were statistically analysed with a Student's T-Test for unpaired data. P=n.s.

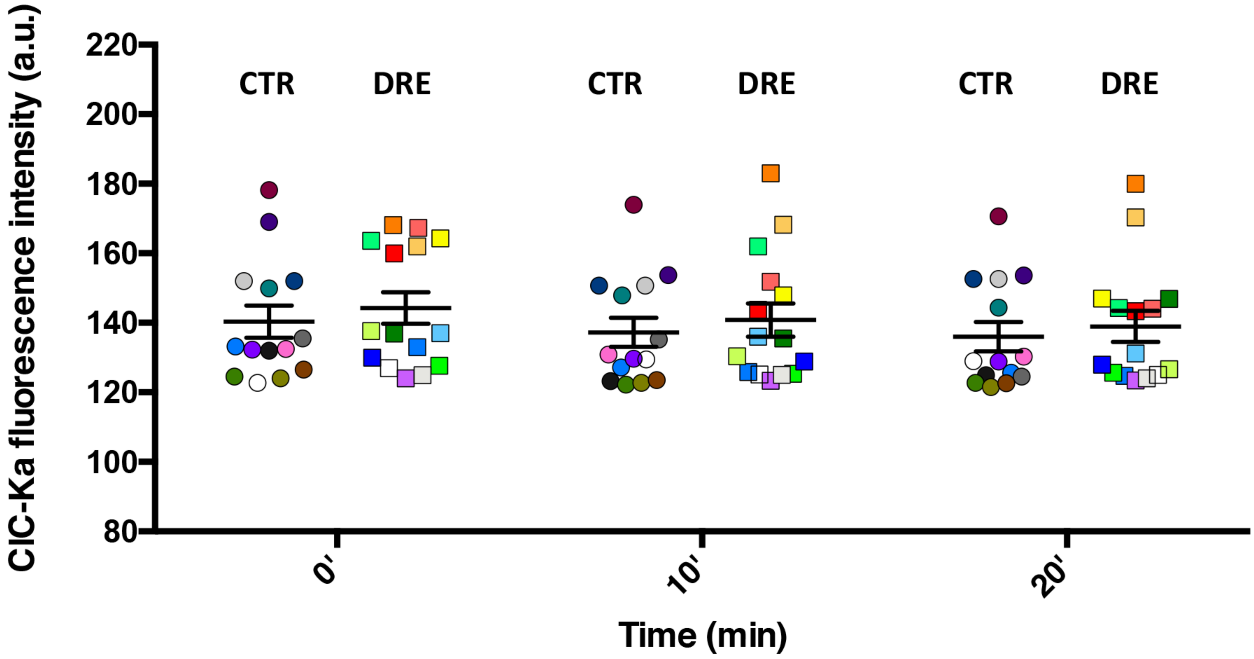

Supplementary Figure 3: Region of interest (ROI) for GFP-ClC-Ka fluorescence quantification where selected at time 0 where the channel colocalizes with WGA. The same ROI was used for the subsequent time points. ClC-Ka fluorescence intensity values are plotted for each cell analyzed in function of the three time points. Circles indicate cells perfused continuously with Ringer's solution (n=14), squares indicate cells perfused with DRE after time 0 (n=15). Each color indicates a single cell analyzed for the three time points. Statistical analysis was performed with one-way ANOVA (Tukey's multiple comparison test). P=n.s.

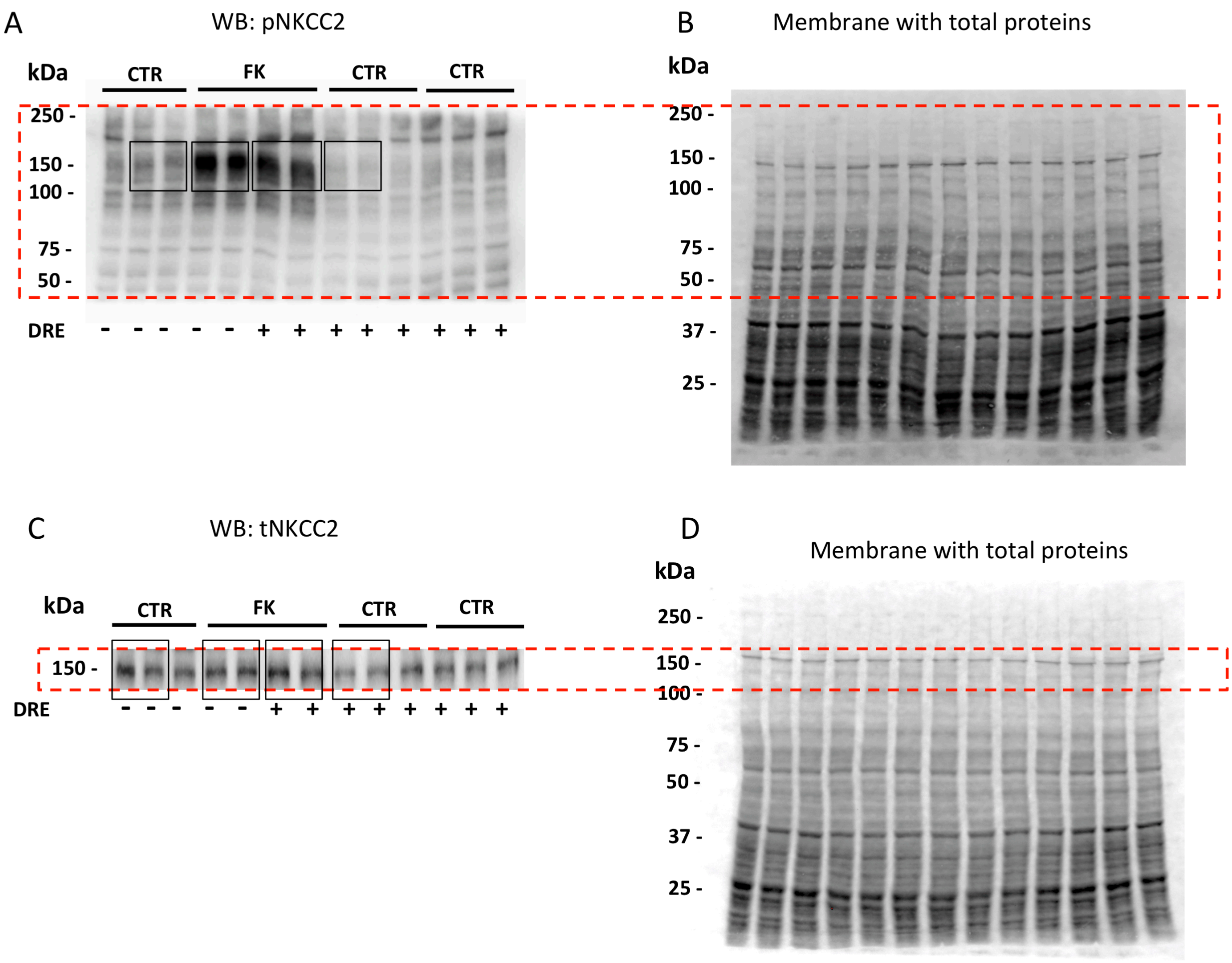

Supplementary Figure 4. NKCC2 expression analysis with stain free precast 7.5 % gels. A) Representative immunoblot for phospho-NKCC2 (pNKCC2) in lysates from mouse kidney slices in control conditions (CTR) or stimulated with Forskolin (FK) in the presence of DRE where indicated (+,- DRE). B) Total protein detection in the correspondent membrane by Stain-Free (SF) technology. The red dotted box indicates the cropped portion of the membrane incubated with the primary antibody. Black boxes indicate the parts of the blot shown in the Supplementary Figure 1A for pNKCC2 western blotting. C) Representative immunoblot for total NKCC2 (tNKCC2) in lysates from mouse kidney slices in control conditions (CTR) or stimulated with Forskolin (FK) in the presence of DRE where indicated (+,- DRE). D) Total protein detection in the correspondent membrane by Stain-Free (SF) technology. The red dotted box indicates the cropped portion of the membrane incubated with the primary antibody. Black boxes indicate the parts of the blot shown in Supplementary Figure 1A for tNKCC2 western blotting.

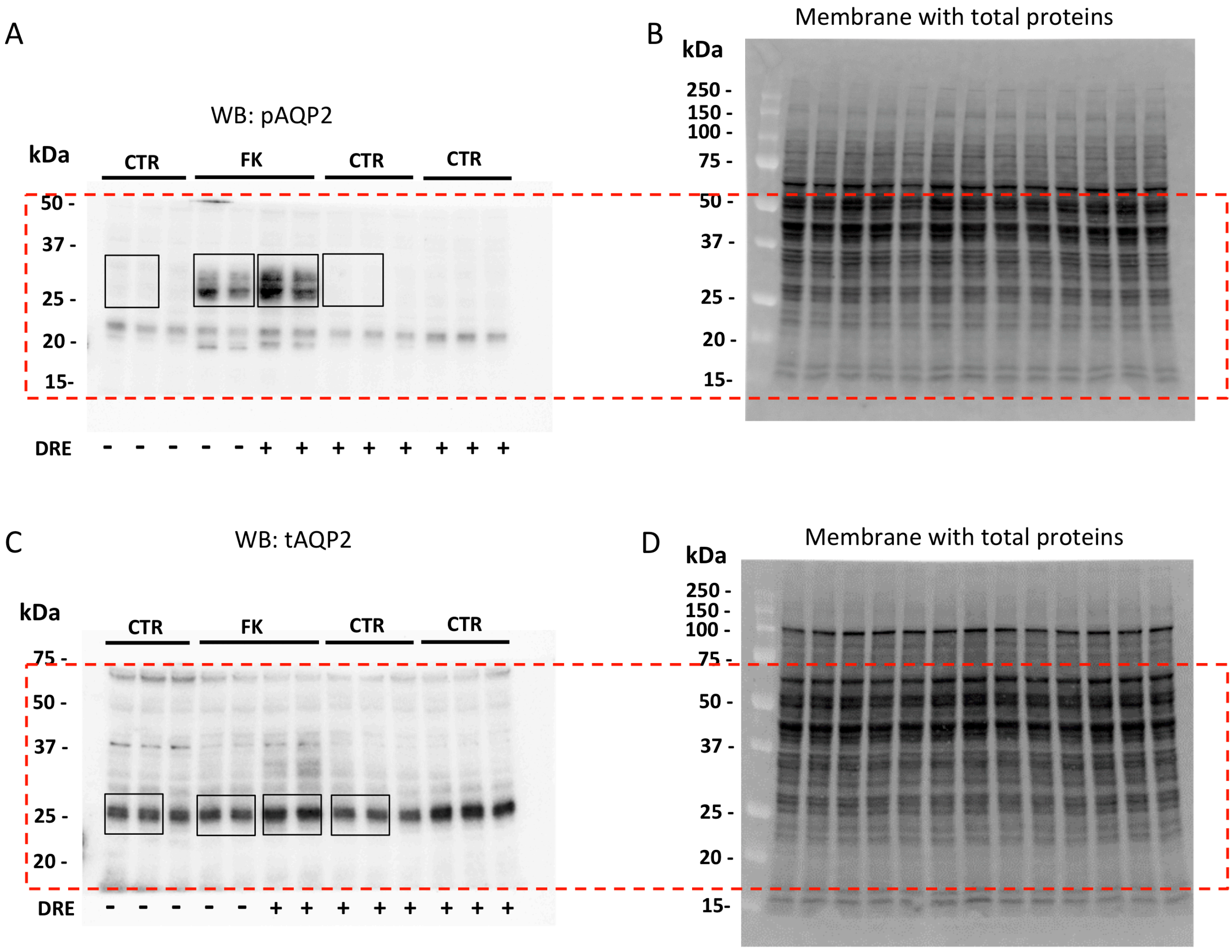

Supplementary Figure 5. AQP2 expression analysis in mouse kidney slices with stain free precast 12 % gels. A) Representative immunoblot for phospho-AQP2 (pAQP2) in lysates from mouse kidney slices in control conditions (CTR) or stimulated with Forskolin (FK) in the presence of DRE where indicated (+,- DRE). B) Total protein detection in the correspondent membrane by Stain-Free (SF) technology. The red dotted box indicates the cropped portion of the membrane incubated with the primary antibody. Black boxes indicate the parts of the blot shown in the Supplementary Figure 1B for pAQP2 western blotting. C) Representative immunoblot for total AQP2 (tAQP2) in lysates from mouse kidney slices in control conditions (CTR) or stimulated with Forskolin (FK) in the presence of DRE where indicated (+,- DRE). D) Total protein detection in the correspondent membrane by Stain-Free (SF) technology. Red dotted box indicates a cropped portion of the membrane incubated with the primary antibody. Black boxes indicate the parts of the blot shown in the Supplementary Figure 1B for tAQP2 western blotting.
